# Implications of Stemness Features in 1059 Hepatocellular Carcinoma Patients from Five Cohorts: Prognosis, Treatment Response, and Identification of Potential Compounds

**DOI:** 10.3390/cancers14030563

**Published:** 2022-01-23

**Authors:** Haoming Mai, Haisheng Xie, Mengqi Luo, Jia Hou, Jiaxuan Chen, Jinlin Hou, De-ke Jiang

**Affiliations:** State Key Laboratory of Organ Failure Research, Guangdong Key Laboratory of Viral Hepatitis Research, Guangdong Institute of Liver Diseases, Department of Infectious Diseases and Hepatology Unit, Nanfang Hospital, Southern Medical University, Guangzhou 510515, China; celadon@i.smu.edu.cn (H.M.); philovo@i.smu.edu.cn (H.X.); qml21910144@i.smu.edu.cn (M.L.); hj22010244@i.smu.edu.cn (J.H.); chenjiaxuan56@smu.edu.cn (J.C.)

**Keywords:** stemness, hepatocellular carcinoma, mRNAsi, prognosis, treatment response, OCLR

## Abstract

**Simple Summary:**

Cancer stemness has been reported to drive hepatocellular carcinoma (HCC) tumorigenesis and treatment resistance. However, comprehensive interpretations of transcriptomic stemness features in HCC patients have not been conducted in multiple cohorts. Our aim was to interpret clinical and therapeutic implications of transcriptional stemness features and explore potential compounds for HCC treatment. We found that transcriptional stemness indexes (mRNAsi) were independently associated with worse HCC prognosis. The HCC stemness risk model (HSRM) developed in this study significantly predicted prognosis and treatment response in various HCC cohorts. Analysis of two stemness subtypes suggested several liver-specific metabolic pathways, and mutations of TP53 and RB1 were associated with HCC transcriptional stemness. Moreover, we also identified potential compounds that target HCC transcriptional stemness. Our findings comprehensively characterized transcriptional stemness as a risk factor in HCC progression and treatment.

**Abstract:**

Cancer stemness has been reported to drive hepatocellular carcinoma (HCC) tumorigenesis and treatment resistance. In this study, five HCC cohorts with 1059 patients were collected to calculate transcriptional stemness indexes (mRNAsi) by the one-class logistic regression machine learning algorithm. In the TCGA-LIHC cohort, we found mRNAsi was an independent prognostic factor, and 626 mRNAsi-related genes were identified by Spearman correlation analysis. The HCC stemness risk model (HSRM) was trained in the TCGA-LIHC cohort and significantly discriminated overall survival in four independent cohorts. HSRM was also significantly associated with transarterial chemoembolization treatment response and rapid tumor growth in HCC patients. Consensus clustering was conducted based on mRNAsi-related genes to divide 1059 patients into two stemness subtypes. On gene set variation analysis, samples of subtype I were found enriched with pathways such as DNA replication and cell cycle, while several liver-specific metabolic pathways were inhibited in these samples. Somatic mutation analysis revealed more frequent mutations of *TP53* and *RB1* in the subtype I samples. In silico analysis suggested topoisomerase, cyclin-dependent kinase, and histone deacetylase as potential targets to inhibit HCC stemness. In vitro assay showed two predicted compounds, Aminopurvalanol-a and NCH-51, effectively suppressed oncosphere formation and impaired viability of HCC cell lines, which may shed new light on HCC treatment.

## 1. Introduction

Liver cancer is one of the most common cancers worldwide [1]. Globally, an estimated 906,000 new cases of liver cancer and 830,000 deaths attributable to liver cancer were reported in 2020 [1]. Liver cancer is considered one of the most lethal cancers, with a 5 year survival rate of just 18% [2]. Hepatocellular carcinoma (HCC) accounts for a vast majority of all cases of liver cancer (75–85%) [1]. Tumor resection and ablation are the main strategies for treatment of HCC patients at an early stage (BCLC stage 0 or A); however, ideal candidates for these strategies are relatively limited [3,4]. Moreover, owing to the insidious onset of HCC, the majority of patients are diagnosed at an advanced stage [3,4]. For patients with intermediate-stage tumors (BCLC stage B), transarterial therapy, especially transarterial chemoembolization (TACE), is considered as a standard treatment option. However, a systematic review showed that only half of HCC patients (52.5%) show objective response with TACE [5]. In addition, systemic therapies are recommended for patients with intermediate- and advanced-stage tumors [4]. Despite substantial improvement in the efficacy of HCC systemic therapies, most HCC patients have a dismal prognosis [6,7,8,9]. Therefore, further research on tumorigenesis and treatment of HCC is a key imperative.

Stemness refers to the self-renewal and differentiation potential of normal stem cells [10,11]. Previous studies have reported a subset of cells, known as cancer stem cells (CSCs), that acquire stem-cell-like characteristics and play an important role in the tumorigenesis and progression of various cancers [12,13]. CSCs are more likely to drive cancer metastasis, recurrence, and therapeutic resistance than other tumor cells [14]. In the context of HCC, such CSCs may also equip tumors with various characteristics to fit in the tumor microenvironment and acquire chemoresistance, though the CSCs model in HCC has been heavily debated [15,16,17,18]. The dedifferentiated cells in HCC may also arise from adult hepatocytes, which could dedifferentiate into progenitor-like precursor cells that ultimately transform into HCC [17]. These findings implicated that the oncogenic dedifferentiation of HCC may derive from various sources. In addition, many studies have also shown that cancer stemness of HCC is associated with dysregulation of pathways such as hypoxia, Wnt, Notch, and NF-κB signaling pathways [19,20,21,22]. This suggests that cancer stemness of HCC may be driven by a transcriptional network that comprises multiple altered molecular pathways.

The one-class logistic regression (OCLR) machine learning algorithm was proved as a robust method to quantify the cancer stemness by two different indices that used the pluripotent stem cell samples of a PCBC data set to train stemness signatures based on mRNA expression or DNA methylation and, subsequently, applied them to score tumor samples [23,24]. The mRNA expression-based stemness index (mRNAsi) is reflective of gene expression profile and mDNAsi was reflective of epigenetic characteristics [23]. Several studies have validated the potential of applying mRNAsi in various tumors by integrating multiple data sets to explore the clinical and biological implications of the transcriptomic stemness feature [25,26,27]. Therefore, systematic assessment of such a transcriptomic stemness index in multiple cohorts may facilitate in-depth characterization of HCC. Moreover, pharmacogenomic data sets and multiple liver cancer cell line models have also been used to screen novel compounds for HCC treatment [28]. Meanwhile, cancer stemness has been reported to induce resistance of HCC to different drugs [29,30,31]. Considering the above facts, it is worthy of screening compounds targeting HCC cancer stemness by leveraging the HCC stemness index and public pharmacogenomic model.

In the current study, we included data of 1059 HCC patients belonging to five independent cohorts to calculate mRNAsi of HCC samples using the OCLR machine learning algorithm. The main findings of this research were summarized in Table S1. Briefly, we developed an HCC stemness risk model (HSRM) to discriminate overall survival (OS) in the TCGA-LIHC cohort and validated its efficacy in four independent HCC cohorts. The model was also found to be a potential indicator of rapid tumor growth and TACE treatment response of HCC patients. Based on mRNAsi-related genes, we then conducted consensus clustering to divide these HCC patients into two stemness subtypes with distinct functional annotations, genomic variations, and clinical features. Finally, Connectivity Map (CMap) and the Liver Cancer Model Repository (LIMORE) were used to screen anti-stemness compounds. NCH-51 and Aminopurvalanol-a were selected to validate their effects by in vitro assay.

## 2. Materials and Methods

### 2.1. Data Collection from HCC Cohorts

Five HCC cohorts (i.e., TCGA-LIHC [32], LIRI-JP [33], CHCC-HBV [34], GSE14520 [35], and GSE54236 [36]) were included in the current study. Transcriptome data of TCGA-LIHC, LIRI-JP, and CHCC-HBV were RNA sequencing data, while transcriptome data of GSE14520 and GSE54236 were microarray data.

Data from the TCGA-LIHC cohort, including transcriptome, somatic mutation, and clinical data, were downloaded from the Genomic Data Commons (GDC) (https://portal.gdc.cancer.gov/, accessed on 20 May 2021).

Data from the LIRI-JP cohort, including transcriptome, somatic mutation, and clinical data, were downloaded from the International Cancer Genome Consortium (ICGC) portal (https://dcc.icgc.org/projects/LIRI-JP, accessed on 20 May 2021).

Transcriptome data from the Chinese HCC patients with hepatitis B virus infection (CHCC-HBV) cohort were downloaded from The National Omics Data Encyclopedia (NODE) (https://www.biosino.org/node, accessed on 20 May 2021). Clinical data from the CHCC-HBV cohort was obtained from the Supplementary Materials of reference [34].

Data from the GSE14520 (GPL3921) and GSE54236 cohorts, including transcriptome and clinical data, were downloaded from the Gene Expression Omnibus (GEO) (https://www.ncbi.nlm.nih.gov/geo/, accessed on 20 May 2021).

After excluding samples for which complete OS information was not available, we finally enrolled 1059 HCC patients from five cohorts, and their information is summarized in Table 1. The “ComBat” algorithm of R package “sva” was utilized to correct batch effects from non-biological technical biases [37].

### 2.2. Calculation of the Transcriptional Stemness Index (mRNAsi)

The transcriptomic data of human 229 cell samples from the Progenitor Cell Biology Consortium (PCBC) (https://www.synapse.org/, accessed on 26 May 2021) were downloaded by the R package “synapser”. The OCLR algorithm was used to calculate the mRNAsi, a previously reported machine learning algorithm, based on transcriptomic data of human stem (78 samples) and non-stem cells (151 samples) [23,24]. The R package “gelnet” was used to train the stemness signatures, and it was applied to score HCC samples from five cohorts.

### 2.3. Classification of HCC Patients Based on mRNAsi-Related Genes

To identify mRNAsi-related genes in the TCGA-LIHC cohort, correlations between gene expression levels and mRNAsi were assessed using Spearman correlation coefficients; |R| > 0.5 was considered as the threshold for determining mRNAsi-related genes.

By using the R package “ConsensusClusterPlus”, unsupervised consensus clustering was conducted to classify 1059 HCC patients from five HCC cohorts based on the expression of mRNAsi-related genes [38]. By sampling 80% of the data in each iteration, the clustering was performed with 1000 iterations. The consensus heatmap, the proportion of ambiguous clustering (PAC) algorithm, and area under the cumulative distribution function (CDF) curves were used to comprehensively identify the optimal number of categories [39].

To identify differentially enriched Kyoto Encyclopedia of Genes and Genomes (KEGG) pathways of the stemness subtypes, gene set variation analysis (GSVA) was performed by the “GSVA” R package [40]. The enrichment scores of KEGG pathways were compared between the two stemness subtypes using the R package “limma”, and a Benjamini–Hochberg false discovery rate (FDR) < 0.05 and |log2FC| > 0.35 were used as cut off values to identify the most differentially enriched KEGG pathways [41].

The somatic mutation data from the LIHC-TCGA (MAF format, 353 patients) and LIRI-JP (simple somatic mutation format, 229 patients) cohorts were downloaded to compare the somatic mutations between the two stemness subtypes. The frequencies and types of mutations were analyzed and visualized by the R package “Maftools” [42].

### 2.4. Construction and Validation of the HSRM

Transcriptome data and OS information from the TCGA-LIHC cohort were used to construct the HSRM. To identify prognostic hub genes among mRNAsi-related genes, least absolute shrinkage and selection operator (LASSO) regression analysis was performed using the R package “glmnet” [43]. Then, HSRM was constructed by multivariate Cox regression analysis based on these prognostic hub genes. The LIRI-JP, CHCC-HBV, GSE14520, and GSE54236 cohorts were used to test the predictive efficiency of HSRM by Kaplan–Meier plots and time-dependent receiver operating characteristic (ROC) curve analysis.

Transcriptome data and treatment response information from the GSE104580 cohort were used to test the efficacy of HSRM in discriminating responders and non-responders to TACE treatment. Transcriptome data and calculated tumor volume doubling time of the GSE54236 cohort were used to explore the association between HSRM and rapid tumor growth in HCC patients.

### 2.5. CMap Analysis

The CMap database (https://clue.io/, accessed on 1 July 2021) was used to screen potential compounds targeting the molecular pathways and genes associated with the stemness features of HCC patients. A list of differentially expressed genes (DEGs) were used to query the CMap database. Based on the median value of the mRNAsi, 1059 HCC patients from five cohorts were divided into high and low mRNAsi groups. By using the R package “limma”, DEGs between the high and low mRNAsi groups were selected with an FDR < 0.05 and |fold change (FC)| > 1 as cut off values. For functional annotation of the DEGs, enrichment analysis was conducted to identify significant KEGG pathways using the R package “clusterprofiler”. Gene set enrichment analysis (GSEA) was conducted to identify significant differentially enriched KEGG pathways between the high and low mRNAsi groups using the R package “clusterprofiler”. The CMap connectivity score is a standardized measure ranging from −100 to 100. In the present study, compounds with a connectivity score ≤ −95 were identified as potential compounds and sorted by shared mechanisms of action (MOAs).

### 2.6. Cell Lines

The HCC cell lines, Huh7 and Hep3B, were obtained from the Cell Bank of the Chinese Academy of Sciences (Shanghai, China). All cell lines were maintained in Dulbecco’s modified Eagle’s medium (DMEM) supplemented with 10% fetal bovine serum (FBS) at 37 °C in an incubator with 5% CO_2_.

### 2.7. Cell Viability Assay

Cell counting kit-8 (CCK-8) assay was conducted according to the manufacturer’s protocol. Huh7 or Hep3B cells were seeded in 96-well plates (Corning Inc., Corning, NY, USA) at a density of 1000 cells per well. Dimethylsulfoxide (DMSO) and different concentrations of Aminopurvalanol-a (1, 2, 4, 8, and 16 µM) and NCH-51 (0.8, 1.6, 3.2, 6.4, and 12.8 µM) were added after 24 h. The plate was incubated for additional 24, 48, 72, 96, and 120 h. Then, 10 μL CCK-8 reagent was added into each well, and the absorbance value was measured at a wavelength of 450 nm after incubation for 2 h at 37 °C. All experiments were performed in triplicate.

### 2.8. Oncosphere Formation Assay

Huh7 or Hep3B cells were seeded in an ultra-low attachment 6-well plate (Corning Inc.) at a density of 4000 cells per well in the presence of Aminopurvalanol-a (2, 4, and 8 µM), NCH-51 (1.6, 3.2, and 6.4 µM), and DMSO. DMEM F12 (Invitrogen, Waltham, MA, USA) was supplemented with 20 ng/mL basic fibroblast growth factor (bEGF) (Invitrogen), 20 ng/mL epidermal growth factor (EGF) (Invitrogen), and B27 (Invitrogen). These cells were incubated at 37 °C with 5% CO_2_ incubator for 1–2 weeks, and spheres larger than 100 µm were counted using ImageJ. All experiments were performed in triplicate.

### 2.9. Statistical Analysis

All statistical analyses were performed using the R statistical software (version 4.0). Between-group differences were assessed using the Wilcoxon test. Multi-group comparisons were performed using the Kruskal–Wallis test. Spearman’s correlation test was used to assess correlation between two continuous variables. Contingency table variables were analyzed by Chi-squared test or Fisher’s exact test. Survival analysis was performed by the Kaplan–Meier method using the R package “survival”. Time-dependent receiver ROC curves analysis was performed by the R package “timeROC”. Cox regression models (univariate or multivariate) were established using the R package “survival”. To determine independent prognostic factors for overall survival (OS), Cox regression methods were used and factors with a *p* < 0.2 in univariate analyses were kept for multivariate analyses. The median value was used as the cut off to classify HCC patients into a high mRNAsi group and a low mRNAsi group in Kaplan–Meier survival analysis and Cox regression analysis. The median value was used as the cut off to classify HCC patients into a high HSRM group and a low HSRM group in Kaplan–Meier survival analysis. Differential analysis was conducted via the R package “limma”. The accuracy of predictive models was evaluated by the area under the curve (AUC). *p*-Values < 0.05 were considered indicative of statistical significance.

## 3. Results

### 3.1. Association of Transcriptional Stemness Index with Clinical and Molecular Features

**Figure 1:**
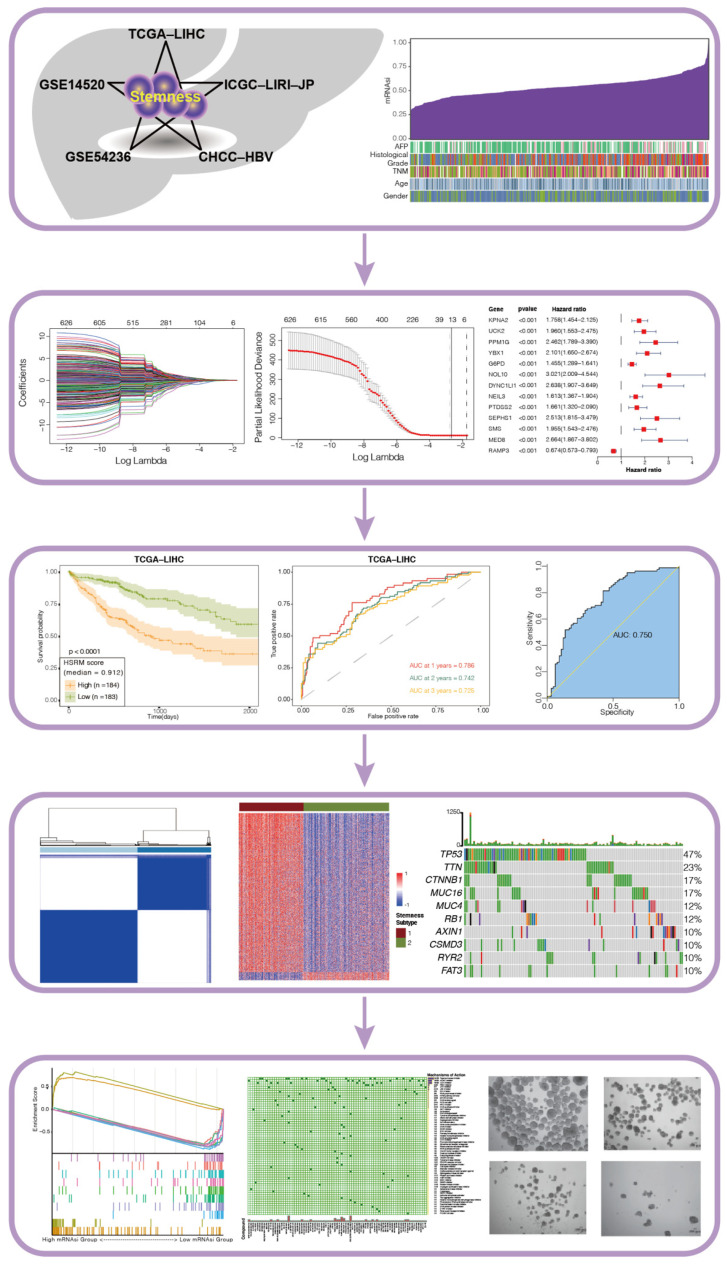
Schematic illustration of the study’s design.

As shown in Figure 1, five HCC cohorts with complete OS information and transcriptomic data were collected for systematic analysis of stemness (Table 1). The mRNAsi of HCC samples was calculated using the OCLR machine learning algorithm (Supplementary Materials Table S2).

**Figure 2:**
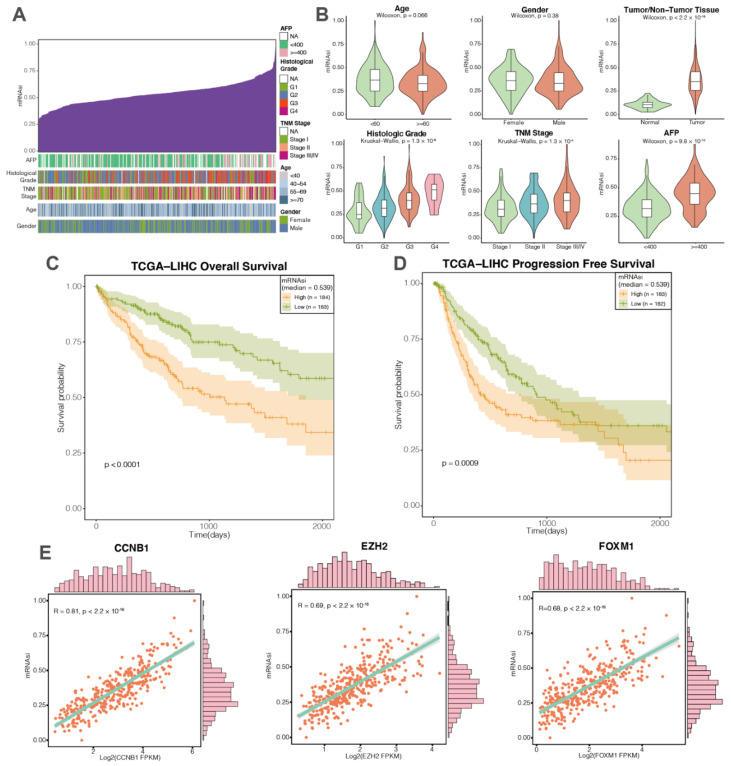
Clinical and molecular features associated with the mRNAsi in HCC patients. (**A**) Overview of the association between mRNAsi and clinical features of HCC patients. Columns represent samples sorted by mRNAsi. Rows represent clinical features associated with mRNAsi. (**B**) Violin plots of mRNAsi of the HCC patients classified by age, sex, histological grade, TNM stage, and AFP levels. The mRNAsi of tumor tissues and paratumoral normal tissues are also displayed. (**C**,**D**) High mRNAsi correlated with significantly poor overall survival (OS) and recurrence-free survival (RFS) (log-rank test, *p* < 0.0001 and *p* = 0.0009, respectively). (**E**) Positive association of mRNAsi with several well-characterized cancer stemness regulators (i.e., CCNB1, FOXM1, and EZH2).

We first investigated the association of mRNAsi with various clinical features in the TCGA-LIHC cohort (Figure 2A). We observed no significant association of mRNAsi with age or sex of the HCC patients (Figure 2B). High mRNAsi was associated with advanced histological grade and TNM stage. HCC patients with a high level of alpha fetal protein (AFP; biomarker of HCC) had a significantly higher mRNAsi level (Figure 2B). In addition, primary tumor of HCC patients showed significantly higher mRNAsi compared with paratumoral normal tissues (Figure 2B). We then explored the prognostic relevance of mRNAsi in HCC patients using Kaplan–Meier analysis. HCC patients with higher mRNAsi scores showed significantly worse OS and progression-free survival (PFS) (Figure 2C,D).

As a transcriptional index, mRNAsi is reflective of gene expressions associated with HCC stemness [23]. We thus assessed the correlation between mRNAsi and gene expression levels, among which CCNB1 was the most mRNAsi-associated gene (Figure 2E). In addition, expression levels of several well-characterized cancer stemness regulators, such as FOXM1 and EZH2, also showed a strong correlation with mRNAsi among the HCC patients of the TCGA-LIHC cohort (Figure 2E) [44,45].

### 3.2. Training and Validation of HSRM of HCC Stemness in Five HCC Cohorts

In the TCGA-LIHC cohort, we found mRNAsi might be a risk factor of shorter OS in HCC patients (Figure 2C). We then evaluated prognostic factors for OS of HCC patients in the TCGA-LIHC cohort by Cox regression methods. In the univariate analysis, HCC OS was statistically associated with the pathological stage, tumor stage, and mRNAsi (Table 2, *p* < 0.05). Multivariate Cox regression analysis showed that mRNAsi was significantly associated with HCC OS (Table 2, *p* < 0.05). These results suggested that mRNAsi was independently associated with OS in HCC patients.

Then, the TCGA-LIHC cohort was used to train the HSRM, which was subsequently validated in other four independent cohorts (Table 1). 

**Figure 3:**
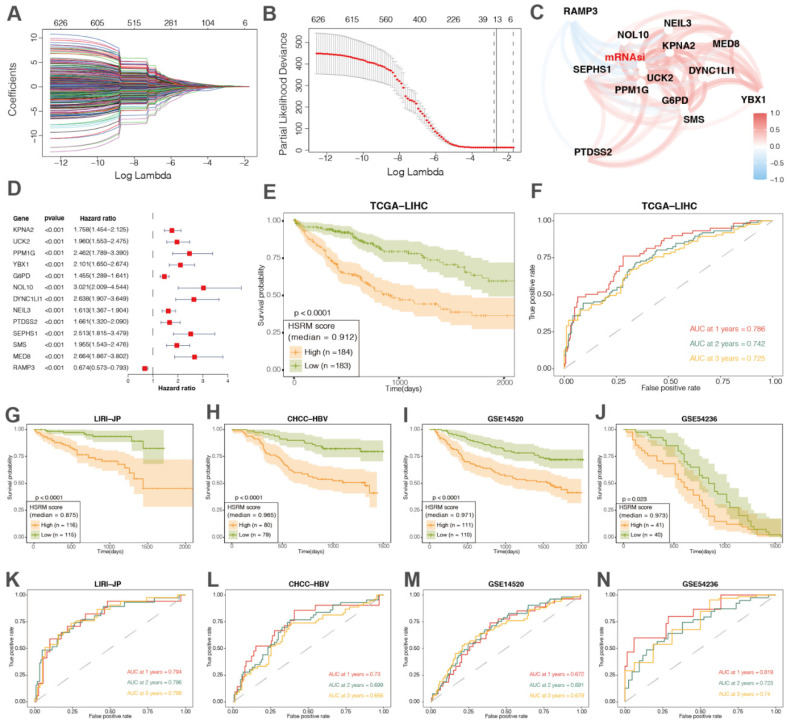
Establishment and validation of a prognostic HCC stemness risk model (HSRM) for HCC patients: (**A**,**B**) LASSO algorithm was used to identify the most robust prognostic genes among mRNAsi-related genes; (**C**) correlation network involving the 13 genes and mRNAsi in the training (TCGA-LIHC) cohort; (**D**) forest plot showing the hazard ratio of each prognostic gene; (**E**) Kaplan–Meier analysis: HCC patients with higher HSRM scores had significantly worse OS in the training (TCGA-LIHC) cohort; (**F**) time-dependent receiver operating characteristic (ROC) curves demonstrate the clinical significance of HSRM in predicting the 1 year, 2 year, and 3 year OS in the training (TCGA-LIHC) cohort. Validation of the prognostic value of HSRM in four independent cohorts by Kaplan–Meier analysis (**G**–**J**) and time-dependent ROC (**K**–**N**).

As the training set, we first identified 626 genes as mRNAsi-related genes (|R| > 0.5) based on the mRNAsi and transcriptome data of TCGA-LIHC cohorts. Then, 13 prognostic hub genes were identified from 626 mRNAsi-related genes using the LASSO regression models (Figure 3A,B). Among these hub genes, only RAMP3 showed a negative correlation with mRNAsi, while the other genes showed a positive correlation with mRNAsi including stem/progenitor cells regulators such as KPNA2 and YBX1 (Figure 3C,D) [46,47,48,49,50]. Then, multivariate Cox regression analysis was conducted to develop the HSRM based on the expression levels of 13 prognostic hub genes (Figure 3E). The formula for the HSRM was as follows: risk score = −KPNA2 × 0.0426 + UCK2 × 0.1762 − PPM1G × 0.046 + YBX1 × 0.2481 − G6PD × 0.0491 + NOL10 × 0.3045 + DYNC1LI1 × 0.3166 + NEIL3 × 0.1102 + PTDSS2 × 0.2484 + SEPHS1 × 0.2446 − SMS × 0.0027 + MED8 × 0.1280 − RAMP3 × 0.1211 (Table S3). The predictive performance of HSRM in the TCGA-LIHC cohort was evaluated by time-dependent ROC curve analysis; the AUC values of 1 year, 2 year, and 3 year OS were 0.786, 0.742, and 0.725, respectively (Figure 3F and Figure S1A).

Then, we used four independent cohorts (692 patients from different regions) to validate the predictive efficacy of HSRM. In line with the training cohort, HSRM significantly discriminated patients with distinct prognosis (Figure 3G,H), indicating the applicability of this model for HCC induced by various risk factors. Moreover, we also assessed the efficiency of HSRM in each validation cohort by time-dependent ROC curve analysis; the AUC values of 1 year, 2 year, and 3 year OS in these cohorts demonstrated that HSRM could robustly predict the prognosis of HCC patients (Figure 3K–N and Figure S1B–E). Moreover, we also combined HSRM with TNM and the Barcelona Clinic Liver Cancer (BCLC) staging system to improve the discrimination power (Figures S2 and S3). In our results, only BCLC stage may significantly improve the performance of HSRM. Due to the data limitation, TNM and BCLC stage data were not available in every HCC cohort in this study (Table 1); thus, more HCC cohorts should be used to validate these results in the future study.

**Figure 4:**
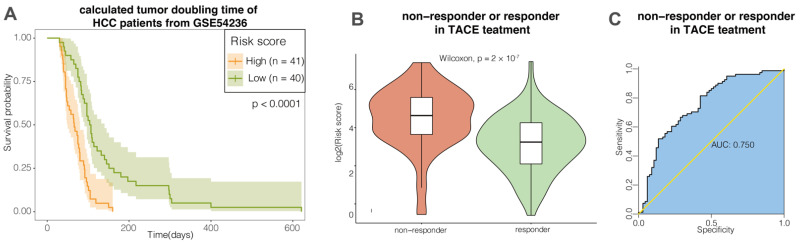
HCC stemness risk model (HSRM) was significantly associated with rapid tumor growth and TACE treatment response among HCC patients: (**A**) Kaplan–Meier analysis—HCC patients with higher HSRM scores had significantly shorter calculated tumor doubling time in the GSE54236 cohort; (**B**) violin plots of mRNAsi of HCC patients classified by response status to TACE treatment; (**C**) ROC curves demonstrate the clinical significance in predicting the response to TACE treatment in the GSE104580 cohort.

Moreover, the tumor volume doubling time of HCC patients from the GSE54236 cohort were calculated based on imaging data [36]. As shown in the Figure 4A, a higher score of HSRM may indicate a faster tumor growth rate in HCC patients. GSE104580 is a cohort publicly available with information pertaining to the therapeutic response to TACE. TACE is considered as part of standard treatment options for HCC [4]. In our results, HSRM significantly predict response to TACE in HCC patients with AUC > 0.7 (Figure 4B,C). These results suggested that in addition to the prognostic relevance, HSRM may also be associated with disease progression and treatment response of HCC patients.

### 3.3. Consensus Clustering Divided HCC Patients into Two Stemness Subtypes with Distinct Functional Annotation and Somatic Mutation Pattern

**Figure 5:**
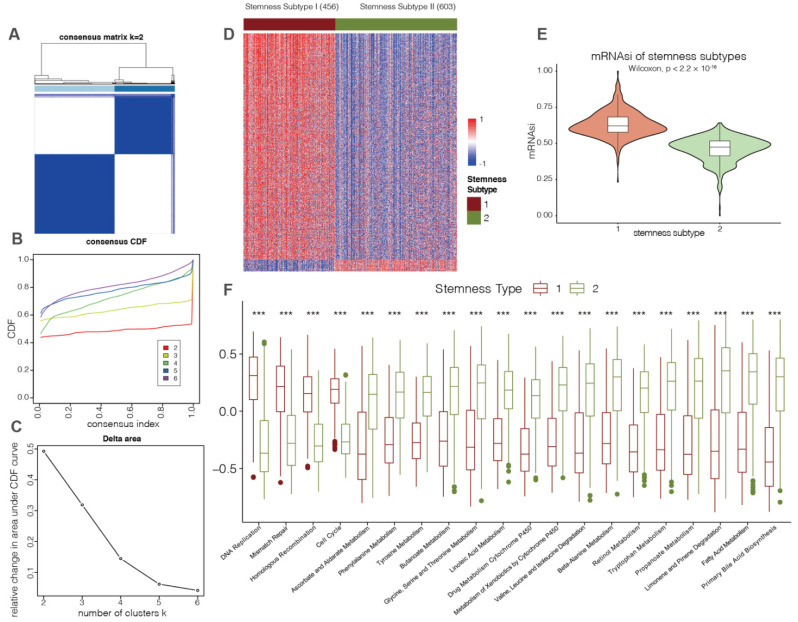
Identification of two HCC stemness subtypes with distinct functional annotations. (**A**–**C**) Consensus clustering was conducted to classify HCC stemness subtypes. Consensus clustering matrix (**A**), cumulative distribution function (**C**–**F**) curves (**B**) and the relative change in the area under the CDF curve (**C**) from k = 2 to 6 was used to determine the optimal cluster number as 2. (**D**) Heatmap showing the expression levels of mRNAsi-related genes between the two HCC stemness subtypes. The color red represents genes that were positively correlated with mRNAsi, and the color blue represents genes that were negatively correlated with mRNAsi. (**E**) Violin plots of the mRNAsi of HCC patients classified by HCC stemness subtypes. (**F**) Boxplots illustrating the KEGG pathways with significantly differential enrichment scores, which were evaluated by GSVA analysis between HCC stemness subtypes (***, *p* ≤ 0.001).

To explore the underlying mechanisms contributing to the HCC stemness, we conducted unsupervised consensus clustering to stratify 1059 HCC patients of five cohorts based on the expression levels of the mRNAsi-related genes mentioned above. According to the consensus heatmap, the PAC algorithm and the CDF curves (k value = 2), two stemness subtypes were identified (Figure 5A–C). We found that subtype I (456 patients, 43%) exhibited higher mRNAsi than subtype II (603 patients, 57%) (Figure 5D,E).

First, GSVA was performed to investigate the functional differences between the two stemness subtypes. As shown in the boxplots, four pathways (i.e., DNA replication, mismatch repair, homologous recombination, and cell cycle) showed a positive correlation with stemness subtype I (Figure 5F). Conversely, a series of metabolic pathways, such as primary bile acid biosynthesis and fatty acid metabolism, were found to be enriched in the HCC samples of subtype II (Figure 5F). These results indicated that the various metabolisms of stemness subtype I tumors might be undermined, which suggests potential links with cancer stemness in HCC.

**Figure 6:**
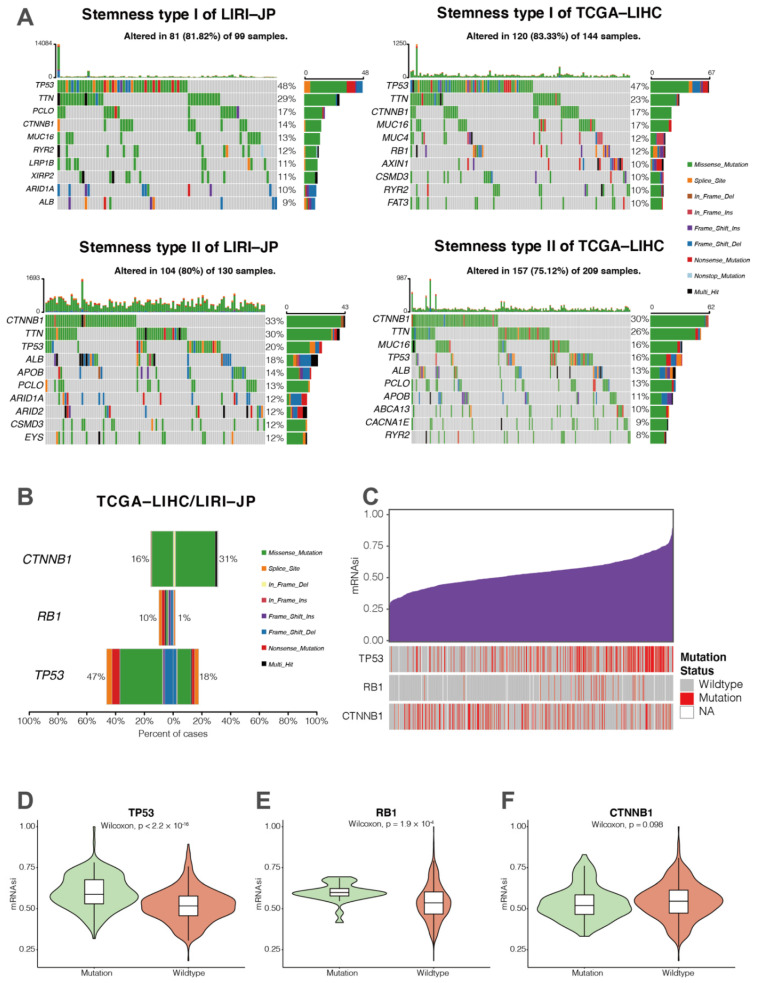
Comparisons of somatic mutations between HCC stemness subtypes. (**A**) Waterfall plots showing the top 10 mutated genes of HCC stemness subtype I (**top**) and II (**bottom**) in the LIRI-JP (**left**) and TCGA-LIHC (**right**) cohorts, respectively. (**B**) Most differentially mutated genes between HCC stemness subtypes in the LIRI-JP and TCGA-LIHC cohorts. (**C**) Overview of the association between mRNAsi and several somatic mutations (i.e., TP53, RB1, and CTNNB1) in HCC patients from the LIRI-JP and TCGA-LIHC cohorts. Columns represent samples sorted by mRNAsi. Rows represent somatic mutations associated with mRNAsi. (**D**–**F**) Violin plots of mRNAsi of HCC patients classified by the mutation status of TP53, RB1, and CTNNB1.

Subsequently, we downloaded the somatic mutation data of 229 and 353 HCC patients from the LIRI-JP and TCGA-LIHC cohorts, respectively. The somatic mutation pattern of each cohort was visualized with the R package “Maftools” (Figure 6A). TP53 was found to be the most frequently mutated gene of stemness subtype I in both cohorts, whereas the CTNNB1 was the most frequently mutated gene of stemness subtype II (Figure 6A). When we pooled data of LIRI-JP and TCGA-LIHC cohorts, only three genes were identified as differentially mutated genes by somatic mutation analysis (*p*-adjust < 0.05, Figure 6B). We found mutation of RB1, a well-known tumor suppressor, was also enriched in tumors of stemness subtype I with a relatively low mutation rate (Figure 6B). To investigate the association between mRNAsi and somatic mutations of TP53, RB1, and CTNNB1, we then compared mRNAsi between wild-type and mutated HCC samples. We found the mRNAsi of TP53 mutant or RB1 mutant HCC samples were significantly higher than wild-type HCC samples, while there was no significant difference between the CTNNB1 wild-type and the CTNNB1 mutant (Figure 6C–E). These findings demonstrate that HCC stemness might be linked with TP53 or RB1 tumor mutations.

### 3.4. Identification of Potential Compounds Targeting Transcriptional Stemness of HCC

**Figure 7:**
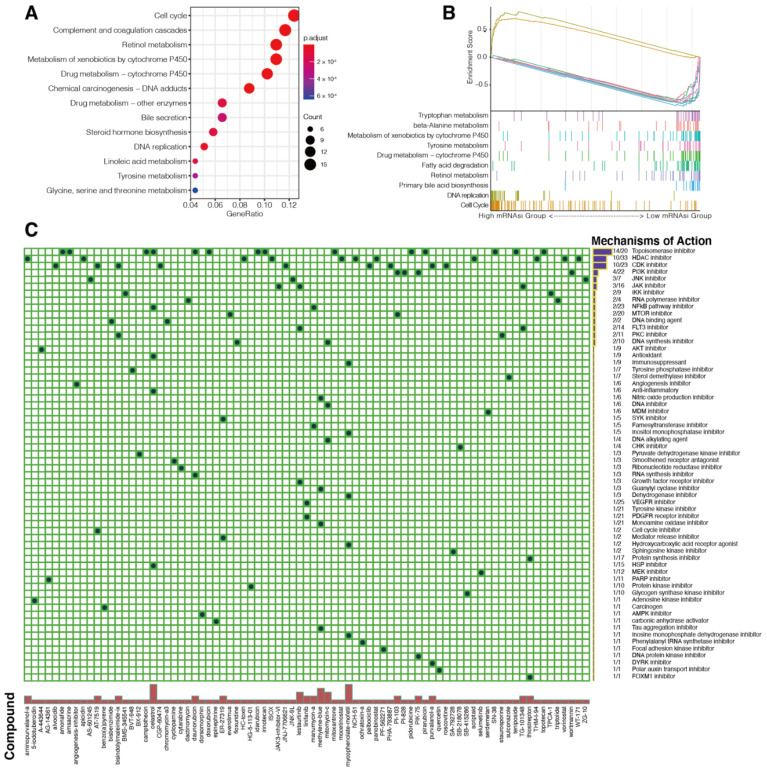
Screening of potential compounds targeting HCC cancer stemness using the cMAP data set. Pathway enrichment analysis (**A**) of DEGs between the high and low mRNAsi groups of 1059 HCC patients. (**B**) GSEA between the high and low mRNAsi groups of 1059 HCC patients. (**C**) Heatmap exhibiting the potential compounds (columns) that share mechanisms of action (MOAs) (rows). All potential compounds had an enrichment score ≤ −95.

In recent years, high-throughput technologies have been applied to investigate the effects of compounds against various cancer types. We first used the OCLR algorithm to calculate mRNAsi for each tumor sample from all 1059 HCC patients from the five cohorts collected in the current study. Then, we identified 209 DEGs from the high mRNAsi and low mRNAsi groups of the HCC samples using the R package “limma”. Consistent with the GSVA between two stemness subtypes, pathway enrichment analysis and GSEA demonstrated the activation of cell cycle pathway and DNA replication pathway in high mRNAsi group, while several metabolic pathways were downregulated in this group (Figure 7A,B). Further, we sought to screen candidate compounds using the 209 DEGs as input in the CMap database, which is a comprehensive data set containing gene expression profiles of multiple cell lines tested by nearly 5000 compounds [51]. Our results identified 81 compounds as potential candidate compounds (Figure 7C). Among these, topoisomerase inhibitors, CDK inhibitors, and HDAC inhibitors were top hits of MOAs, indicating that drugs targeting these molecules may inhibit transcriptional cancer stemness of HCC (Figure 7C).

**Figure 8:**
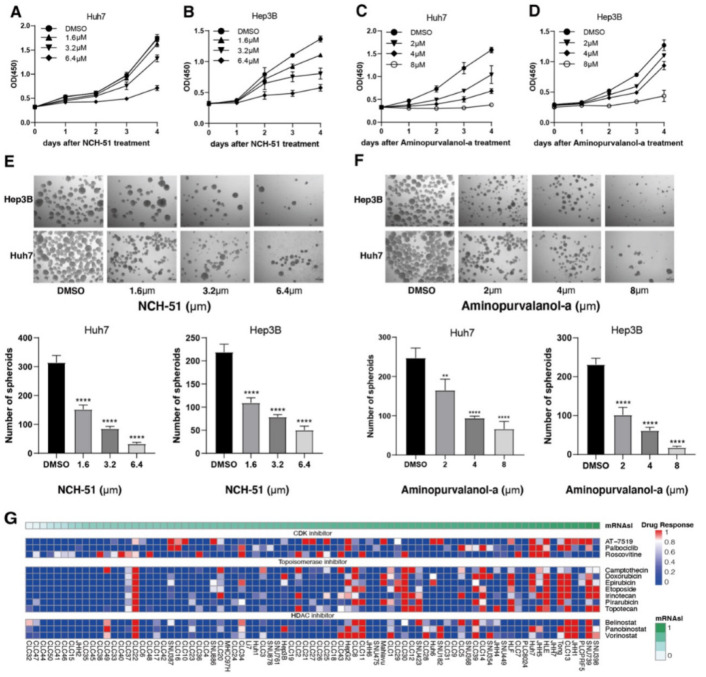
Assessment of the response to potential compounds by in vitro experiment and the LIMORE data set. (**A**–**D**) Results of the CCK8 assay: both Aminopurvalanol-a and NCH-51 effectively suppressed proliferation and viability of Huh7 and Hep3B cells. (**E**, **F**) Results of the. sphere formation assay: sphere forming-ability of Huh7 and Hep3B cells was suppressed by Aminopurvalanol-a and NCH-51 (**, *p* < 0.01; **** *p* < 0.0001). (**G**) Heatmap exhibiting inhibitors of topoisomerase, CDK, and HDAC with drug response in 81 liver cancer cell lines.

We searched experimental and clinical evidence of these compounds in PubMed (https://www.ncbi.nlm.nih.gov/pubmed/, accessed on 4 July 2021) and found all these predicted topoisomerase inhibitors have been researched in HCC treatment (Table S4). Among the unstudied CDK and HDAC inhibitors, Aminopurvalanol-a and NCH-51 had the most significant cMAP scores (Table S4). We thus selected these two compounds to perform in vitro experiments using two commonly used liver cancer cell lines: Huh7 and Hep3B. The results of the CCK8 assay demonstrated that both Aminopurvalanol-a and NCH-51 effectively suppressed proliferation and viability of Huh7 and Hep3B cells (Figure 8A–D). In addition, sphere-forming ability was also inhibited by Aminopurvalanol-a and NCH-51 in both Huh7 and Hep3B cells (Figure 8E,F).

In addition, 13 compounds targeting topoisomerase, CDK, and HDAC were selected to assess their response in 81 liver cancer cell line models in LIMORE, a pharmacogenomics database of liver cancer [28]. As shown in Figure 8G, higher mRNAsi of liver cancer cell lines were associated with a stronger response to these compounds. Further studies are required to verify the potency of these compounds with selective target pathways.

## 4. Discussion

By acquiring stem-cell-like characteristics, the oncogenic dedifferentiation may drive tumorigenesis, metastasis, tumor recurrence and resistance to cancer treatment [12,13,14]. In the current study, transcriptome data of HCC cohorts were used to calculate mRNAsi. Among patients of TCGA-LIHC cohort, higher mRNAsi showed a correlation with higher histological grade, advanced TNM stage, and higher AFP levels. High mRNAsi was also associated with poor prognosis of HCC patients and multivariate Cox regression analysis demonstrated that mRNAsi was an independent prognostic factor for HCC OS. We identified 626 mRNAsi-related genes by assessing the correlation between gene expression levels and mRNAsi scores. Subsequently, we constructed the HSRM with TCGA-LIHC cohort as the training data set and validated it in the other four HCC cohorts. The risk score of HSRM was a robust predictor of OS of HCC patients. In addition, HSRM was also significantly associated with TACE treatment response and rapid tumor growth. Based on the gene expression levels of mRNAsi-related genes, we classified 1059 patients into two stemness subtypes with differential mRNAsi levels. The two stemness subtypes exhibited distinct molecular pathway enrichment and somatic mutation patterns. Moreover, potential compounds targeting cancer stemness of HCC were identified by interrogating the CMap database. Finally, CDK inhibitor (CDKi) Aminopurvalanol-a and HDAC inhibitor (HDACi) NCH-51 were selected to conduct in vitro experiments to further test their potential role in HCC treatment.

In the current study, the OCLR machine learning algorithm was used to assess stemness of HCC tissues. Based on DEGs between CSCs and non-CSCs, several prognostically relevant cancer stemness scores have been developed in the context of various tumors [52,53,54,55,56]. Compared with these studies, the OCLR machine learning algorithm was derived from a more comprehensive gene set and tissue origins [23]. Due to the fact of its flexibility and versatility, the OCLR machine learning algorithm has been used to investigate cancer stemness and relevant gene signatures in various tumor types including prostate cancer glioblastoma, breast cancer, and esophageal cancer [25,26,57,58].

Over the last decade, several prognostic prediction models for HCC patients have been developed based on transcriptomic data [59,60,61]. In the current study, we found mRNAsi was an independent prognostic factor for HCC OS in the TCGA-LIHC cohort. The HSRM developed from the TCGA-LIHC cohort could robustly predict OS in four independent HCC cohorts, indicating the applicability of this model to HCC patients of different races and etiologies. Moreover, previous studies indicated that the CSCs subset and stemness-associated genes may confer resistance to TACE treatment [62,63]. In our study, we observed an association of HSRM with response to TACE treatment. These results prompted us to explore the potential compounds that could target cancer stemness of HCC.

A recent study demonstrated that suppression of a series of liver-specific metabolic modules in HCC and the deficiency of the urea cycle may promote expansion of cancer stem cells [64]. The transcriptomic profile of the two subtypes was analyzed by GSVA, and we found enrichment of a series of metabolic pathways in subtype II, which possessed significant lower mRNAsi levels than subtype I. These results indicated that metabolic pathways, such as primary bile acid biosynthesis and fatty acid metabolism, were intertwined with HCC cancer stemness. *TP53*, a well-known tumor suppressor, is one of the most frequently mutated genes in HCC patients; in addition, *TP53* mutation is associated with the poor prognosis of HCC patients [32]. In a previous study, human pluripotent stem cells were found to recurrently acquire and expand negative *TP53* mutations, which conferred selective advantage during cell culture [65]. In our study, somatic mutation analysis in both the TCGA-LIHC and LIRI-JP cohorts demonstrated significant enrichment of the *TP53* mutation in stemness subtype I. The mRNAsi levels of HCC patients with mutant *TP53* were also significantly higher than the mRNAsi levels of other HCC patients.

In this study, we also explored the infiltration levels of immune cells in HCC samples using different algorithms. However, there was no solid evidence of any strong correlation between HCC cancer stemness and immune infiltration. Although we observed significant association between stemness subtypes and infiltration level of several immune cells, the infiltration difference was relatively weak (Figure S4). These results were consistent with a previous study that found a weak correlation of cancer stemness with anticancer immunity and mutation load in HCC [66].

Previous studies showed stemness of HCC contributing to sorafenib and TACE treatment resistance [67,68], and controlling stemness may help to increase their treatment response. As mRNAsi is a stemness index derived from transcriptional features, we identified potential compounds and mechanisms that may reduce mRNAsi transcriptionally by interrogating the cMAP database. Topoisomerase, CDK, and HDAC inhibitors ranked as the top three mechanisms in the MOA analysis, in which 34 compounds were identified. We also found a strong association between mRNAsi of HCC cell lines and the response of compounds targeting topoisomerase, CDK, and HDAC in the LIMORE data set. These molecules were closely associated with cell cycle and DNA replication, which constitutively contribute to stem cell self-renewal [69,70,71].

Sorafenib remained as standard of care for advanced HCC for a decade since its approval in 2007. After, the combination of atezolizumab and bevacizumab was the first treatment option that prolonged OS of advanced HCC. During this period, novel CDKi and HDACi have been continuously developed for treatment of HCC [72,73,74], and a series of clinical trials were undertaken to overcome sorafenib resistance [75,76,77]. Similarly, clinical trials (i.e., NCT03024437 and NCT03280563) are being conducted to explore whether CDKi and HDACi have a synergistic antitumor effect in combination with atezolizumab and bevacizumab in other tumor types. These studies suggest the potential for combinations of HCC first-line therapies with novel CDKi or HDACi, which may provide clinical benefits for more patients. At present, there is no evidence of the efficacy of Aminopurvalanol-a (CDKi) and NCH51 (HDACi) in the HCC treatment. To widen the spectrum of candidate compounds for HCC treatment, Aminopurvalanol-a and NCH-51 were selected to conduct in vitro experiments.

However, further studies are required to overcome the limitations of current research. First, dedifferentiated cells may arise from bona fide stem and progenitor cells or by dedifferentiation from non-stem cancer cells. It is not clear whether the transcriptional stemness index derived from bulk tumor samples is representative of these rare cell populations [23,66,78]. Second, more samples from larger multi-center HCC cohorts should be used to validate the predictive efficacy of the HSRM, especially for treatment responses. Third, further studies are required to explore the experimental and clinical value of Aminopurvalanol-a and NCH51 in HCC treatment.

## 5. Conclusions

We systemically evaluated the stemness features in five independent cohorts of HCC patients and deciphered the associations between molecular pathways, somatic variations, and HCC stemness subtypes. The HSRM showed stable performance in discriminating the OS of HCC patients. We screened potential compounds to target HCC cancer stemness and further studies are warranted to explore their therapeutic value.

## Figures and Tables

**Table 1:** Demographical features of the HCC cohorts used in this study.

Cohort Names	Experiment Type	Number of Patients	Region	Age (Mean ± SD)	Gender	
					Male	Female
TCGA-LIHC	Illumina HiSeq RNAseqV2	367	The United States	59.67 ± 13.33	248	119
LIRI-JP	Illumina HiSeq	231	Japan	67.30 ± 10.13	170	61
CHCC-HBV	Illumina HiSeq X Ten	159	China	53.69 ± 10.90	128	31
GSE14520	Affymetrix Human Genome-U133A	221	China	50.82 ± 10.62	191	30
GSE54236	Agilent-014850 Whole Human Genome Microarray 4x44K G4112F	81	Italy	Not reported	64	17

**Table 2:** Prognostic factors of overall survival in the TCGA-LIHC cohort.

Prognostic Factor	Univariate Analysis			Multivariate Analysis		
	HR	95% CI	*p* *	HR	95% CI	*p* *
Sex (female vs. male)	0.809	0.568–1.152	0.239			
Age (<60 vs. ≥60)	0.831	0.586–1.178	0.298			
Child Pugh Grade (B/C vs. A)	1.63	0.804–3.303	0.175	1.757	0.834–3.699	0.138
Histologic Grade (G2-4 vs. G1)	1.207	0.733–1.989	0.459			
Pathological Stage (II–IV vs. I)	2.016	1.383–2.939	<0.0001	1.671	0.202–13.809	0.634
T stage (II–IV vs. I)	2.054	1.435–2.942	<0.0001	1.072	0.13–8.85	0.949
Vascular Tumor Invasion (Macro/Micro vs. None)	1.306	0.863–1.975	0.206			
Inflammation Extent Type of Adjacent Tissue (Severe/Mild vs. none)	1.177	0.726–1.908	0.508			
AFP (≥400 vs. < 400 ng/mL)	1.05	0.643–1.716	0.845			
mRNAsi (high vs. low) ^a^	1.912	1.348- 2.717	<0.0001	1.873	1.138–3.077	0.014

HR, hazard ratio; CI, confidence interval; AFP, alpha fetal protein. ^a^ The median value was used as the cut off to classify HCC patients into a high mRNAsi group and a low mRNAsi group; * Wald test.

## Data Availability

Data from the TCGA-LIHC cohort are available in the GDC repository (https://portal.gdc.cancer.gov/, accessed on 20 May 2021). Data from the LIRI-JP cohort are available in the ICGC portal (https://dcc.icgc.org/projects/LIRI-JP, accessed on 20 May 2021). The CHCC-HBV cohort is available in the NODE repository (https://dcc.icgc.org/projects/LIRI-JP, accessed on 20 May 2021). Clinical data of CHCC-HBV cohort is available in Supplementary Materials files from Reference [33]. Data from the GSE14520 (GPL3921), GSE54236, and GSE104580 cohorts are available in the GEO repository (https://www.ncbi.nlm.nih.gov/geo/, accessed on 20 May 2021). The transcriptomic data of 229 human cell samples are available in the PCBC repository (https://www.synapse.org/, accessed on 26 May 2021). Drug responses data and transcriptomes of 81 liver cancer cell line models are available from the LIMORE website (https://www.picb.ac.cn/limore/, accessed on 26 June 2021).

## Supplementary Materials

The following supporting information can be downloaded at: https://www.mdpi.com/article/10.3390/cancers14030563/s1, Figure S1: Time-dependent receiver operating characteristic (ROC) curves demonstrating the clinical significance of HSRM in predicting the OS in five HCC cohorts; Figure S2: Time-dependent receiver operating characteristic (ROC) curves to compare the prognostic accuracy of HSRM with TNM stage in predicting the OS in four HCC cohorts. TNM stages were transformed into numeric codes before they were entered into the COX regression analysis. Numeric codes are as follows: stage I = 1, stage II = 2, stage III = 3, and stage IV = 4; Figure S3: Time-dependent receiver operating characteristic (ROC) curves to compare the prognostic accuracy of HSRM with the Barcelona Clinic Liver Cancer (BCLC) staging system in predicting the OS in two HCC cohorts. BCLC stages were transformed into numeric codes before they were entered into the COX regression analysis. Numeric codes are as follows: stage 0 = 0, stage 1 = 1, stage 2 = 2, and stage 3 = 3; Figure S4: Deconvolution algorithms Cibersort and ssGSEA of 27 knowledge-based functional gene expression signatures (Fges) were used to compare immune infiltration between stemness subtype I and II of HCC patients in five cohorts; Table S1: Summary of the main findings in current research; Table S2: Stemness index (mRNAsi) of 1059 HCC patients from five cohorts; Table S3: Thirteen HSRM hub genes selected by LASSO regression models; Table S4: Topoisomerase inhibitors, CDK inhibitors, and HDAC inhibitors predicted to transcriptionally inhibit cancer stemness of HCC.

## References

[1] Sung H., Ferlay J., Siegel R.L., Laversanne M., Soerjomataram I., Jemal A., Bray F. (2021). Global cancer statistics 2020: GLOBOCAN estimates of incidence and mortality worldwide for 36 cancers in 185 countries. CA Cancer J. Clin..

[2] Jemal A., Ward E., Johnson C., Cronin K., Ma J., Ryerson B., Mariotto A., Lake A., Wilson R., Sherman R. (2017). Annual Report to the Nation on the Status of Cancer, 1975–2014, Featuring Survival. J. Natl. Cancer Inst..

[3] Roayaie S., Jibara G., Tabrizian P., Park J., Yang J., Yan L., Schwartz M., Han G., Izzo F., Chen M. (2015). The role of hepatic resection in the treatment of hepatocellular cancer. Hepatology.

[4] Villanueva A. (2019). Hepatocellular Carcinoma. N. Engl. J. Med..

[5] Lencioni R., de Baere T., Soulen M., Rilling W., Geschwind J. (2016). Lipiodol transarterial chemoembolization for hepatocellular carcinoma: A systematic review of efficacy and safety data. Hepatology.

[6] Simon T., Duberg A., Aleman S., Chung R., Chan A., Ludvigsson J. (2020). Association of Aspirin with Hepatocellular Carcinoma and Liver-Related Mortality. N. Engl. J. Med..

[7] Bomze D., Meirson T., Azoulay D. (2020). Atezolizumab and Bevacizumab in Hepatocellular Carcinoma. N. Engl. J. Med..

[8] Llovet J., Ricci S., Mazzaferro V., Hilgard P., Gane E., Blanc J., de Oliveira A., Santoro A., Raoul J., Forner A. (2008). Sorafenib in advanced hepatocellular carcinoma. N. Engl. J. Med..

[9] Kudo M., Finn R., Qin S., Han K., Ikeda K., Piscaglia F., Baron A., Park J., Han G., Jassem J. (2018). Lenvatinib versus sorafenib in first-line treatment of patients with unresectable hepatocellular carcinoma: A randomised phase 3 non-inferiority trial. Lancet.

[10] Prasetyanti P., Medema J. (2017). Intra-tumor heterogeneity from a cancer stem cell perspective. Mol. Cancer.

[11] Friedmann-Morvinski D., Verma I. (2014). Dedifferentiation and reprogramming: Origins of cancer stem cells. EMBO Rep..

[12] Ge Y., Gomez N., Adam R., Nikolova M., Yang H., Verma A., Lu C., Polak L., Yuan S., Elemento O. (2017). Stem Cell Lineage Infidelity Drives Wound Repair and Cancer. Cell.

[13] Bjerkvig R., Tysnes B., Aboody K., Najbauer J., Terzis A. (2005). Opinion: The origin of the cancer stem cell: Current controversies and new insights. Nat. Rev. Cancer.

[14] Seguin L., Desgrosellier J., Weis S., Cheresh D. (2015). Integrins and cancer: Regulators of cancer stemness, metastasis, and drug resistance. Trends Cell Biol..

[15] Lee T., Guan X., Ma S. (2021). Cancer stem cells in hepatocellular carcinoma—From origin to clinical implications. Nat. Rev. Gastroenterol. Hepatol..

[16] Craig A., von Felden J., Garcia-Lezana T., Sarcognato S., Villanueva A. (2020). Tumour evolution in hepatocellular carcinoma. Nat. Rev. Gastroenterol. Hepatol..

[17] Sia D., Villanueva A., Friedman S.L., Llovet J.M. (2017). Liver Cancer Cell of Origin, Molecular Class, and Effects on Patient Prognosis. Gastroenterology.

[18] Mu X., Español-Suñer R., Mederacke I., Affò S., Manco R., Sempoux C., Lemaigre F., Adili A., Yuan D., Weber A. (2015). Hepatocellular carcinoma originates from hepatocytes and not from the progenitor/biliary compartment. J. Clin. Investig..

[19] Cui C., Wong C., Kai A., Ho D., Lau E., Tsui Y., Chan L., Cheung T., Chok K., Chan A. (2017). SENP1 promotes hypoxia-induced cancer stemness by HIF-1α deSUMOylation and SENP1/HIF-1α positive feedback loop. Gut.

[20] Lo R., Leung C., Chan K., Ho D., Wong C., Lee T., Ng I. (2018). Cripto-1 contributes to stemness in hepatocellular carcinoma by stabilizing Dishevelled-3 and activating Wnt/β-catenin pathway. Cell Death Differ..

[21] Khosla R., Rastogi A., Ramakrishna G., Pamecha V., Mukhopadhyay A., Vasudevan M., Sarin S., Trehanpati N. (2017). EpCAM+ Liver Cancer Stem-Like Cells Exhibiting Autocrine Wnt Signaling Potentially Originate in Cirrhotic Patients. Stem Cells Transl. Med..

[22] Wang R., Li Y., Tsung A., Huang H., Du Q., Yang M., Deng M., Xiong S., Wang X., Zhang L. (2018). iNOS promotes CD24CD133 liver cancer stem cell phenotype through a TACE/ADAM17-dependent Notch signaling pathway. Proc. Natl. Acad. Sci. USA.

[23] Malta T.M., Sokolov A., Gentles A.J., Burzykowski T., Poisson L., Weinstein J.N., Kaminska B., Huelsken J., Omberg L., Gevaert O. (2018). Machine Learning Identifies Stemness Features Associated with Oncogenic Dedifferentiation. Cell.

[24] Sokolov A., Paull E., Stuart J. (2016). One-class detection of cell states in tumor subtypes. Pac. Symp. Biocomput..

[25] Wang Z., Wang Y., Yang T., Xing H., Wang Y., Gao L., Guo X., Xing B., Wang Y., Ma W. (2021). Machine learning revealed stemness features and a novel stemness-based classification with appealing implications in discriminating the prognosis, immunotherapy and temozolomide responses of 906 glioblastoma patients. Brief. Bioinform..

[26] Zhang C., Chen T., Li Z., Liu A., Xu Y., Gao Y., Xu D. (2021). Depiction of tumor stemlike features and underlying relationships with hazard immune infiltrations based on large prostate cancer cohorts. Brief. Bioinform..

[27] Stahl D., Knoll R., Gentles A.J., Vokuhl C., Buness A., Gutgemann I. (2021). Prognostic Gene Expression, Stemness and Immune Microenvironment in Pediatric Tumors. Cancers.

[28] Qiu Z., Li H., Zhang Z., Zhu Z., He S., Wang X., Wang P., Qin J., Zhuang L., Wang W. (2019). A Pharmacogenomic Landscape in Human Liver Cancers. Cancer Cell.

[29] Ma X., Hu B., Tang W., Xie S., Ren N., Guo L., Lu R. (2020). CD73 sustained cancer-stem-cell traits by promoting SOX9 expression and stability in hepatocellular carcinoma. J. Hematol. Oncol..

[30] Govaere O., Wouters J., Petz M., Vandewynckel Y., Van den Eynde K., Van den Broeck A., Verhulst S., Dollé L., Gremeaux L., Ceulemans A. (2016). Laminin-332 sustains chemoresistance and quiescence as part of the human hepatic cancer stem cell niche. J. Hepatol..

[31] Guan D., Shi J., Zhang Y., Zhao J., Long L., Chen T., Zhang E., Feng Y., Bao W., Deng Y. (2015). Sorafenib enriches epithelial cell adhesion molecule-positive tumor initiating cells and exacerbates a subtype of hepatocellular carcinoma through TSC2-AKT cascade. Hepatology.

[32] The Cancer Genome Atlas Research Network (2017). Comprehensive and Integrative Genomic Characterization of Hepatocellular Carcinoma. Cell.

[33] Fujimoto A., Furuta M., Totoki Y., Tsunoda T., Kato M., Shiraishi Y., Tanaka H., Taniguchi H., Kawakami Y., Ueno M. (2016). Whole-genome mutational landscape and characterization of noncoding and structural mutations in liver cancer. Nat. Genet..

[34] Gao Q., Zhu H., Dong L., Shi W., Chen R., Song Z., Huang C., Li J., Dong X., Zhou Y. (2019). Integrated Proteogenomic Characterization of HBV-Related Hepatocellular Carcinoma. Cell.

[35] Roessler S., Long E., Budhu A., Chen Y., Zhao X., Ji J., Walker R., Jia H., Ye Q., Qin L. (2012). Integrative genomic identification of genes on 8p associated with hepatocellular carcinoma progression and patient survival. Gastroenterology.

[36] Villa E., Critelli R., Lei B., Marzocchi G., Camma C., Giannelli G., Pontisso P., Cabibbo G., Enea M., Colopi S. (2016). Neoangiogenesis-related genes are hallmarks of fast-growing hepatocellular carcinomas and worst survival. Results from a prospective study. Gut.

[37] Leek J., Johnson W., Parker H., Jaffe A., Storey J. (2012). The sva package for removing batch effects and other unwanted variation in high-throughput experiments. Bioinformatics.

[38] Wilkerson M., Hayes D. (2010). ConsensusClusterPlus: A class discovery tool with confidence assessments and item tracking. Bioinformatics.

[39] Șenbabaoğlu Y., Michailidis G., Li J. (2014). Critical limitations of consensus clustering in class discovery. Sci. Rep..

[40] Hänzelmann S., Castelo R., Guinney J. (2013). GSVA: Gene set variation analysis for microarray and RNA-seq data. BMC Bioinform..

[41] Ritchie M., Phipson B., Wu D., Hu Y., Law C., Shi W., Smyth G. (2015). limma powers differential expression analyses for RNA-sequencing and microarray studies. Nucleic Acids Res..

[42] Mayakonda A., Lin D.C., Assenov Y., Plass C., Koeffler H.P. (2018). Maftools: Efficient and comprehensive analysis of somatic variants in cancer. Genome Res..

[43] Friedman J., Hastie T., Tibshirani R. (2010). Regularization Paths for Generalized Linear Models via Coordinate Descent. J. Stat. Softw..

[44] Yao S., Fan L., Lam E. (2018). The FOXO3-FOXM1 axis: A key cancer drug target and a modulator of cancer drug resistance. Semin. Cancer Biol..

[45] Kim K., Roberts C. (2016). Targeting EZH2 in cancer. Nat. Med..

[46] Umemura Y., Koike N., Matsumoto T., Yoo S., Chen Z., Yasuhara N., Takahashi J., Yagita K. (2014). Transcriptional program of Kpna2/Importin-α2 regulates cellular differentiation-coupled circadian clock development in mammalian cells. Proc. Natl. Acad. Sci. USA.

[47] Kwon E., Todorova K., Wang J., Horos R., Lee K., Neel V., Negri G., Sorensen P., Lee S., Hentze M. (2018). The RNA-binding protein YBX1 regulates epidermal progenitors at a posttranscriptional level. Nat. Commun..

[48] Zou F., Tu R., Duan B., Yang Z., Ping Z., Song X., Chen S., Price A., Li H., Scott A. (2020). Drosophila YBX1 homolog YPS promotes ovarian germ line stem cell development by preferentially recognizing 5-methylcytosine RNAs. Proc. Natl. Acad. Sci. USA.

[49] Alkrekshi A., Wang W., Rana P., Markovic V., Sossey-Alaoui K. (2021). A comprehensive review of the functions of YB-1 in cancer stemness, metastasis and drug resistance. Cell. Signal..

[50] Yasuhara N., Yamagishi R., Arai Y., Mehmood R., Kimoto C., Fujita T., Touma K., Kaneko A., Kamikawa Y., Moriyama T. (2013). Importin alpha subtypes determine differential transcription factor localization in embryonic stem cells maintenance. Dev. Cell.

[51] Subramanian A., Narayan R., Corsello S., Peck D., Natoli T., Lu X., Gould J., Davis J., Tubelli A., Asiedu J. (2017). A Next Generation Connectivity Map: L1000 Platform and the First 1,000,000 Profiles. Cell.

[52] Gentles A., Plevritis S., Majeti R., Alizadeh A.J.J. (2010). Association of a leukemic stem cell gene expression signature with clinical outcomes in acute myeloid leukemia. JAMA.

[53] Ng S., Mitchell A., Kennedy J., Chen W., McLeod J., Ibrahimova N., Arruda A., Popescu A., Gupta V., Schimmer A. (2016). A 17-gene stemness score for rapid determination of risk in acute leukaemia. Nature.

[54] Eppert K., Takenaka K., Lechman E., Waldron L., Nilsson B., van Galen P., Metzeler K., Poeppl A., Ling V., Beyene J. (2011). Stem cell gene expression programs influence clinical outcome in human leukemia. Nat. Med..

[55] Ben-Porath I., Thomson M., Carey V., Ge R., Bell G., Regev A., Weinberg R. (2008). An embryonic stem cell-like gene expression signature in poorly differentiated aggressive human tumors. Nat. Genet..

[56] Pinto J., Kalathur R., Oliveira D., Barata T., Machado R., Machado S., Pacheco-Leyva I., Duarte I., Futschik M. (2015). StemChecker: A web-based tool to discover and explore stemness signatures in gene sets. Nucleic Acids Res..

[57] Huang R., Li Z., Zhang J., Zeng Z., Zhang J., Li M., Wang S., Xian S., Xue Y., Chen X. (2020). Construction of Bone Metastasis-Specific Regulation Network Based on Prognostic Stemness-Related Signatures in Breast Invasive Carcinoma. Front. Oncol..

[58] Yi L., Huang P., Zou X., Guo L., Gu Y., Wen C., Wu G. (2020). Integrative stemness characteristics associated with prognosis and the immune microenvironment in esophageal cancer. Pharmacol. Res..

[59] Chaudhary K., Poirion O.B., Lu L., Garmire L.X. (2018). Deep Learning-Based Multi-Omics Integration Robustly Predicts Survival in Liver Cancer. Clin. Cancer Res..

[60] Yang C., Huang X., Li Y., Chen J., Lv Y., Dai S. (2021). Prognosis and personalized treatment prediction in TP53-mutant hepatocellular carcinoma: An in silico strategy towards precision oncology. Brief. Bioinform..

[61] Kim J.H., Sohn B.H., Lee H.S., Kim S.B., Yoo J.E., Park Y.Y., Jeong W., Lee S.S., Park E.S., Kaseb A. (2014). Genomic predictors for recurrence patterns of hepatocellular carcinoma: Model derivation and validation. PLoS Med..

[62] Wei X., Zhao L., Ren R., Ji F., Xue S., Zhang J., Liu Z., Ma Z., Wang X., Wong L. (2021). MiR-125b Loss Activated HIF1α/pAKT Loop, Leading to Transarterial Chemoembolization Resistance in Hepatocellular Carcinoma. Hepatology.

[63] Lai J., Conley A., Knudsen B., Guindi M. (2015). Hypoxia after transarterial chemoembolization may trigger a progenitor cell phenotype in hepatocellular carcinoma. Histopathology.

[64] Wu T., Luo G., Lian Q., Sui C., Tang J., Zhu Y., Zheng B., Li Z., Zhang Y., Zhang Y. (2021). Discovery of a CPS1-deficient HCC subtype with therapeutic potential via integrative genomic and experimental analysis. Hepatology.

[65] Merkle F.T., Ghosh S., Kamitaki N., Mitchell J., Avior Y., Mello C., Kashin S., Mekhoubad S., Ilic D., Charlton M. (2017). Human pluripotent stem cells recurrently acquire and expand dominant negative P53 mutations. Nature.

[66] Miranda A., Hamilton P.T., Zhang A.W., Pattnaik S., Becht E., Mezheyeuski A., Bruun J., Micke P., de Reynies A., Nelson B.H. (2019). Cancer stemness, intratumoral heterogeneity, and immune response across cancers. Proc. Natl. Acad. Sci. USA.

[67] Xia S., Pan Y., Liang Y., Xu J., Cai X. (2020). The microenvironmental and metabolic aspects of sorafenib resistance in hepatocellular carcinoma. EBioMedicine.

[68] Lanza E., Donadon M., Poretti D., Pedicini V., Tramarin M., Roncalli M., Rhee H., Park Y.N., Torzilli G. (2016). Transarterial Therapies for Hepatocellular Carcinoma. Liver Cancer.

[69] Liu L., Michowski W., Kolodziejczyk A., Sicinski P. (2019). The cell cycle in stem cell proliferation, pluripotency and differentiation. Nat. Cell Biol..

[70] Damelin M., Sun Y.E., Sodja V.B., Bestor T.H. (2005). Decatenation checkpoint deficiency in stem and progenitor cells. Cancer Cell.

[71] Jamaladdin S., Kelly R.D., O’Regan L., Dovey O.M., Hodson G.E., Millard C.J., Portolano N., Fry A.M., Schwabe J.W., Cowley S.M. (2014). Histone deacetylase (HDAC) 1 and 2 are essential for accurate cell division and the pluripotency of embryonic stem cells. Proc. Natl. Acad. Sci. USA.

[72] Lachenmayer A., Toffanin S., Cabellos L., Alsinet C., Hoshida Y., Villanueva A., Minguez B., Tsai H.W., Ward S.C., Thung S. (2012). Combination therapy for hepatocellular carcinoma: Additive preclinical efficacy of the HDAC inhibitor panobinostat with sorafenib. J. Hepatol..

[73] Hsu C., Lin L.I., Cheng Y.C., Feng Z.R., Shao Y.Y., Cheng A.L., Ou D.L. (2016). Cyclin E1 Inhibition can Overcome Sorafenib Resistance in Hepatocellular Carcinoma Cells Through Mcl-1 Suppression. Clin. Cancer Res..

[74] Bollard J., Miguela V., Ruiz de Galarreta M., Venkatesh A., Bian C.B., Roberto M.P., Tovar V., Sia D., Molina-Sánchez P., Nguyen C.B. (2017). Palbociclib (PD-0332991), a selective CDK4/6 inhibitor, restricts tumour growth in preclinical models of hepatocellular carcinoma. Gut.

[75] Bitzer M., Horger M., Ganten T.M., Siveke J.T., Woerns M.A., Dollinger M.M., Gerken G., Scheulen M.E., Wege H., Giannini E.G. (2012). Investigation of the HDAC inhibitor resminostat in patients with sorafenib-resistant hepatocellular carcinoma (HCC): Clinical data from the phase I/II SHELTER study. J. Clin. Oncol..

[76] Bitzer M., Horger M., Giannini E.G., Ganten T.M., Worns M.A., Siveke J.T., Dollinger M.M., Gerken G., Scheulen M.E., Wege H. (2016). Resminostat plus sorafenib as second-line therapy of advanced hepatocellular carcinoma—The SHELTER study. J. Hepatol..

[77] Villa E., Piscaglia F., Geva R., Dalecos G., Papatheodoridis G., Ciomei M., Davite C., Crivori P., Palejwala V., Jacob J. (2020). Phase IIa safety and efficacy of milciclib, a pan-cyclin dependent kinase inhibitor, in unresectable, sorafenib-refractory or -intolerant hepatocellular carcinoma patients. J. Clin. Oncol..

[78] Zheng H., Song K., Fu Y., You T., Yang J., Guo W., Wang K., Jin L., Gu Y., Qi L. (2021). An absolute human stemness index associated with oncogenic dedifferentiation. Brief. Bioinform..

