# Supplementary material for: Implications of Stemness Features in 1059 Hepatocellular Carcinoma Patients from Five Cohorts: Prognosis, Treatment Response, and Identification of Potential Compounds
Source: Cancers (Basel). 2022 Jan 23;14(3):563. doi: 10.3390/cancers14030563 (PMC8833508; doi:10.3390/cancers14030563)
Supplement: Supplementary file 1 [file cancers-14-00563-s001.zip › cancers-1535936-supplementary.pdf]

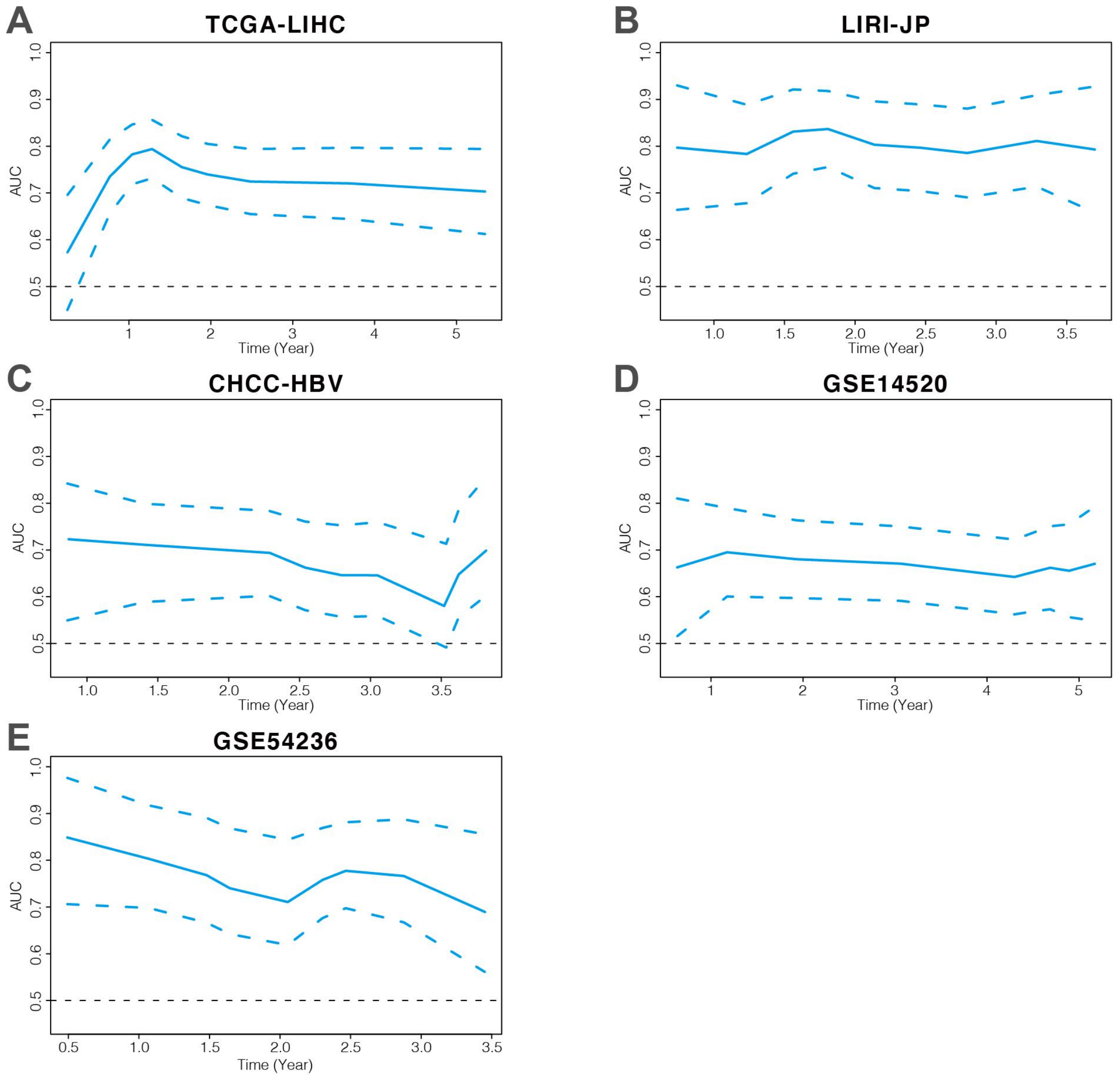

Figure S1. Time-dependent receiver operating characteristic (ROC) curves demonstrate the clinical significance of HSRM in predicting the OS in five HCC cohort.

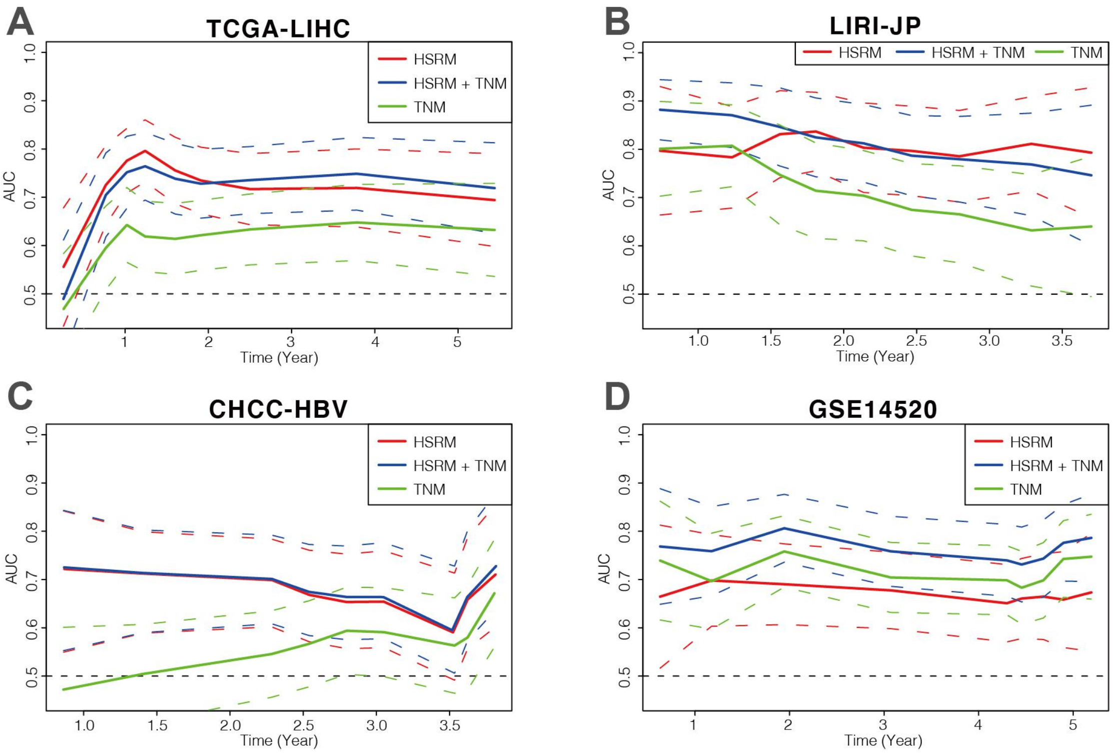

Figure S2. Time-dependent receiver operating characteristic (ROC) curves to compare the prognostic accuracy of HSRM with TNM stage in predicting the OS in four HCC cohorts. TNM stage were transformed into numeric codes before they were entered into the COX regression analysis. Numeric codes are as follows: stage I = 1, stage II = 2, stage III = 3, stage IV = 4 [1].

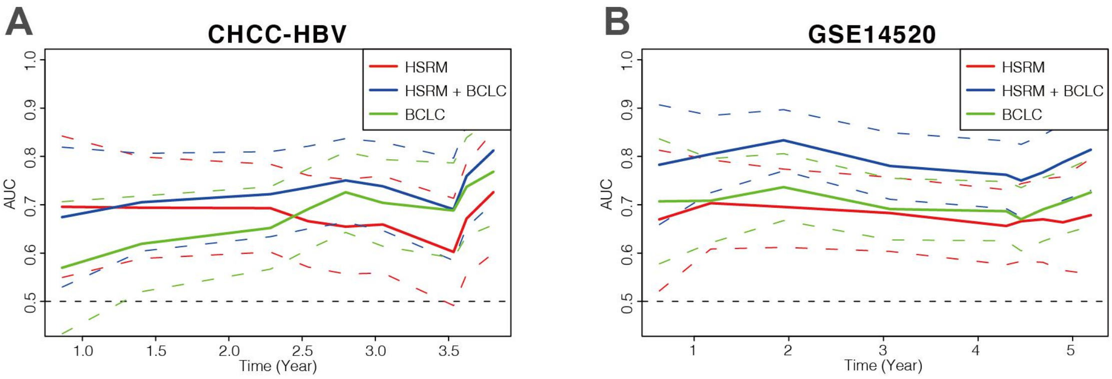

Figure S3. Time-dependent receiver operating characteristic (ROC) curves to compare the prognostic accuracy of HSRM with Barcelona Clinic Liver Cancer (BCLC) staging system in predicting the OS in two HCC cohorts. BCLC stage were transformed into numeric codes before they were entered into the COX regression analysis. Numeric codes are as follows: stage 0 = 0, stage 1 = 1, stage 2 = 2, stage 3 = 3 [1].

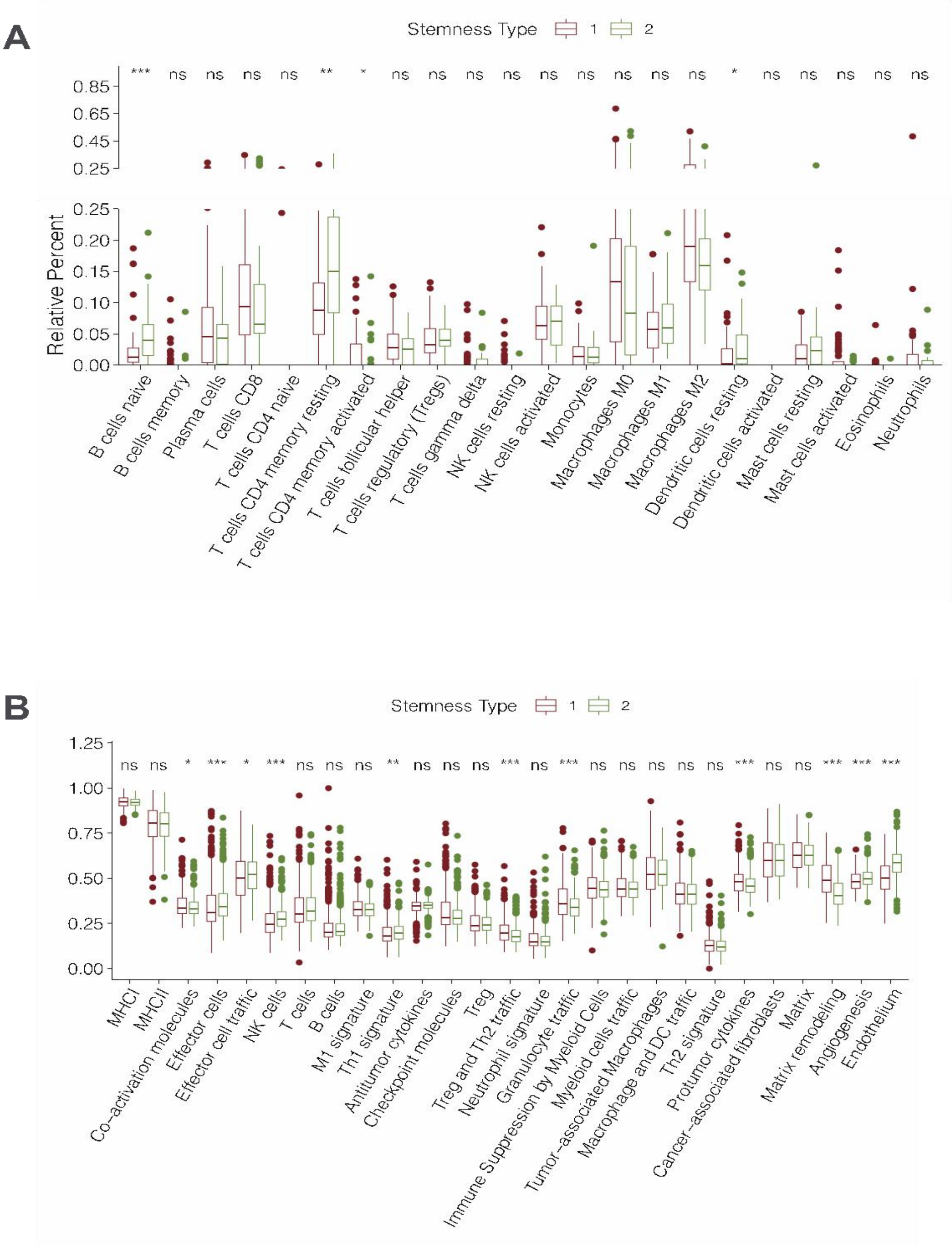

Figure S4. Deconvolution algorithms Cibersort [2] and ssGSEA of 27 knowledge-based functional gene expression signatures (Fges) [3] were used to compare immune infiltration between stemness subtype I and II of HCC patients in five cohorts.

Table S1. Summary of the main findings in current research.

method	datasets	result
Data preprocessing and mRNAsi calculation		
OCLR algorithm	TCGA-LIHC, LIRI-JP, CHCC-HBV, GSE14520, GSE54236	mRNAsi of 1,059 HCC patients
Association of transcriptional stemness index with clinical and molecular features		
Multivariate Cox regression analysis	TCGA-LIHC	mRNAsi might be an independent prognostic factor for HCC patients
Speaman correlation analysis (|R| > 0.5)	TCGA-LIHC	626 mRNAsi-related genes
Training and validation of HSRM of HCC stemness in five HCC cohorts		
LASSO COX analysis, K-M plots, Time-dependent ROC curve analysis	TCGA-LIHC	HSRM was developed from 626 mRNAsi-related genes to predict HCC prognosis
K-M plots, Time-dependent ROC curve analysis	LIRI-JP, CHCC-HBV, GSE14520, GSE54236	HSRM was validated by four indenpendent cohorts
K-M plots	GSE54236	HSRM was significantly associated tumor rapid growth in HCC patients
ROC curve analysis	GSE104580	HSRM was significantly associated with TACE treatment response in HCC patients
Consensus clustering divided HCC patients into two stemness subtypes with distinct functional annotation and somatic mutation pattern		
Unsupervised consensus clustering, GSVA analysis	TCGA-LIHC, LIRI-JP, CHCC-HBV, GSE14520, GSE54236	① 626 mRNAsi-related genes were used to classify 1059 patients into 2 stemness subtypes with distinct mRNAsi level (higher mRNAsi in subtype I); ② Stemness subtype I was enriched with cell cycle, DNA replication pathways, whereas several liver-specific metabolic pathways were inhibited in these

		samples.
Somatic Mutation Analysis	TCGA-LIHC, LIRI-JP	Stemness subtype I was associated with more frequent mutations of TP53 and RB1
Identification of potential compounds targeting transcriptional stemness of HCC		
Enrichment analysis, GSEA, cMAP analysis	TCGA-LIHC, LIRI-JP, CHCC-HBV, GSE14520, GSE54236	① Median value of mRNAsi was used to classify 1059 HCC patients into high mRNAsi and low mRNAsi group; ② Cell cycle and DNA replication pathways associated with higher mRNAsi, whereas several liver-specific metabolic pathways associated with lower mRNAsi; ③ Topoisomerase, cyclin-dependent kinase and histone deacetylase were identified as potential targets to inhibit HCC stemness.
In vitro assay	-	① Aminopurvalanol-a and NCH-51 were selected to conduct *in vitro* assay; ② Aminopurvalanol-a and NCH-51 effectively suppressed oncosphere formation and impaired viability of HCC cell lines.

Table S2. Stemness index (mRNAsi) of 1059 HCC patients from five cohorts.

sample id	mRNAsi	cohort
TCGA.CC.A7II.01A	1	TCGA-LIHC
GSM363129	0.925734669	GSE14520
TCGA.G3.A7M9.01A	0.901653432	TCGA-LIHC
T965	0.89895248	CHCC-HBV
T351	0.896674295	CHCC-HBV
DO45211	0.892884293	LIRI-JP
GSM363070	0.890998196	GSE14520
GSM363245	0.856499246	GSE14520
T385	0.851181961	CHCC-HBV
GSM1310586	0.847352146	GSE54236
TCGA.CC.A5UC.01A	0.845382282	TCGA-LIHC
TCGA.DD.AACP.01A	0.838095021	TCGA-LIHC
T661	0.834034738	CHCC-HBV
DO45127	0.828115909	LIRI-JP
T367	0.823788981	CHCC-HBV
GSM363297	0.8237801	GSE14520
T331	0.822594412	CHCC-HBV
GSM363150	0.813984197	GSE14520
GSM363354	0.809827725	GSE14520
DO45269	0.807479055	LIRI-JP
GSM363083	0.799667173	GSE14520
TCGA.5C.A9VG.01A	0.791367163	TCGA-LIHC
T671	0.791139476	CHCC-HBV
GSM363222	0.78201744	GSE14520
TCGA.CC.A7IE.01A	0.780511254	TCGA-LIHC
DO45145	0.780125992	LIRI-JP
GSM363127	0.775525014	GSE14520
TCGA.RG.A7D4.01A	0.772324419	TCGA-LIHC
TCGA.RC.A6M6.01A	0.769179315	TCGA-LIHC
TCGA.DD.AACL.01A	0.767435807	TCGA-LIHC
DO45259	0.766712119	LIRI-JP
T951	0.760807654	CHCC-HBV
TCGA.G3.AAV6.01A	0.759316952	TCGA-LIHC
DO45275	0.758876698	LIRI-JP
GSM363194	0.757552607	GSE14520
TCGA.DD.A39Y.01A	0.757231125	TCGA-LIHC
TCGA.2Y.A9GY.01A	0.756870161	TCGA-LIHC
TCGA.ED.A7PX.01A	0.755323976	TCGA-LIHC

DO45299	0.753644104	LIRI-JP
DO50815	0.753453579	LIRI-JP
T983	0.753402183	CHCC-HBV
DO45161	0.75106566	LIRI-JP
DO23521	0.750054139	LIRI-JP
TCGA.DD.AA3A.01A	0.749076209	TCGA-LIHC
TCGA.CC.A1HT.01A	0.747616498	TCGA-LIHC
GSM363332	0.743249586	GSE14520
GSM363085	0.742718371	GSE14520
TCGA.CC.A3M9.01A	0.742474592	TCGA-LIHC
T211	0.741779131	CHCC-HBV
TCGA.CC.5258.01A	0.740518183	TCGA-LIHC
GSM363237	0.740072756	GSE14520
T391	0.739041363	CHCC-HBV
TCGA.FV.A4ZQ.01A	0.738799586	TCGA-LIHC
DO50799	0.736086049	LIRI-JP
DO45287	0.734848356	LIRI-JP
TCGA.CC.A8HT.01A	0.734538262	TCGA-LIHC
GSM363268	0.734456685	GSE14520
TCGA.DD.A1EL.01A	0.732612844	TCGA-LIHC
DO45307	0.728613458	LIRI-JP
DO23519	0.726775761	LIRI-JP
GSM363218	0.72532793	GSE14520
T877	0.724349676	CHCC-HBV
T387	0.723872138	CHCC-HBV
GSM363235	0.723757577	GSE14520
TCGA.DD.AAE0.01A	0.722958191	TCGA-LIHC
TCGA.DD.A1EJ.01A	0.721854582	TCGA-LIHC
TCGA.DD.AADC.01A	0.721842499	TCGA-LIHC
TCGA.DD.AACZ.01A	0.721254335	TCGA-LIHC
TCGA.DD.A4NN.01A	0.721130849	TCGA-LIHC
TCGA.BC.A112.01A	0.719214014	TCGA-LIHC
DO45093	0.719034069	LIRI-JP
DO45221	0.718664339	LIRI-JP
DO48730	0.718377173	LIRI-JP
GSM363263	0.718245605	GSE14520
TCGA.DD.AADB.01A	0.717914064	TCGA-LIHC
DO50778	0.717060169	LIRI-JP
GSM363147	0.715472271	GSE14520
DO23525	0.712695383	LIRI-JP

TCGA.CC.A8HS.01A	0.711695954	TCGA-LIHC
GSM363309	0.711087584	GSE14520
T815	0.710877023	CHCC-HBV
T283	0.710795072	CHCC-HBV
T285	0.710046961	CHCC-HBV
TCGA.CC.A5UD.01A	0.707424497	TCGA-LIHC
TCGA.DD.AADN.01A	0.707043541	TCGA-LIHC
GSM363357	0.706850779	GSE14520
GSM363296	0.705984188	GSE14520
DO50806	0.705489458	LIRI-JP
TCGA.DD.AADD.01A	0.704660265	TCGA-LIHC
GSM363350	0.702883568	GSE14520
DO45193	0.702674673	LIRI-JP
GSM1310614	0.702265333	GSE54236
DO23515	0.700996717	LIRI-JP
GSM1310599	0.700393639	GSE54236
DO23526	0.699178075	LIRI-JP
DO48681	0.69875474	LIRI-JP
DO48757	0.698571879	LIRI-JP
DO45267	0.697897449	LIRI-JP
TCGA.BC.A69H.01A	0.69755852	TCGA-LIHC
GSM1310633	0.69509036	GSE54236
T537	0.694618877	CHCC-HBV
DO50785	0.694213195	LIRI-JP
DO45189	0.693353593	LIRI-JP
DO45303	0.692956711	LIRI-JP
DO23537	0.692626599	LIRI-JP
GSM1310576	0.689721111	GSE54236
T743	0.689492744	CHCC-HBV
GSM1310642	0.68655504	GSE54236
GSM363032	0.685444755	GSE14520
DO45305	0.685209293	LIRI-JP
T413	0.685146002	CHCC-HBV
TCGA.CC.A7IG.01A	0.684938486	TCGA-LIHC
DO45185	0.684611106	LIRI-JP
DO48697	0.683834309	LIRI-JP
T463	0.683738976	CHCC-HBV
TCGA.ZP.A9CZ.01A	0.683128976	TCGA-LIHC
TCGA.2Y.A9H0.01A	0.682758066	TCGA-LIHC
DO45209	0.682599668	LIRI-JP

DO48751	0.682337356	LIRI-JP
T411	0.679265537	CHCC-HBV
TCGA.CC.A123.01A	0.67921178	TCGA-LIHC
GSM1310612	0.677421896	GSE54236
GSM363184	0.677132118	GSE14520
GSM363033	0.676340351	GSE14520
TCGA.DD.AADW.01A	0.675585666	TCGA-LIHC
TCGA.CC.5263.01A	0.675025488	TCGA-LIHC
GSM363030	0.675025382	GSE14520
GSM1310573	0.674764922	GSE54236
GSM363077	0.674408907	GSE14520
TCGA.CC.A3MB.01A	0.673621532	TCGA-LIHC
GSM363384	0.673241908	GSE14520
GSM1310603	0.672204962	GSE54236
T567	0.672093648	CHCC-HBV
GSM363386	0.671411077	GSE14520
TCGA.G3.A25T.01A	0.671066335	TCGA-LIHC
GSM363316	0.670674739	GSE14520
T343	0.670361883	CHCC-HBV
DO45265	0.670320301	LIRI-JP
TCGA.DD.A1EF.01A	0.668146189	TCGA-LIHC
TCGA.BC.A3KG.01A	0.66810084	TCGA-LIHC
GSM363011	0.667505473	GSE14520
TCGA.ED.A7PZ.01A	0.667352197	TCGA-LIHC
DO50829	0.665006082	LIRI-JP
GSM362983	0.664825805	GSE14520
GSM363330	0.66402478	GSE14520
T497	0.663719056	CHCC-HBV
TCGA.KR.A7K7.01A	0.662839351	TCGA-LIHC
TCGA.CC.A8HU.01A	0.662170898	TCGA-LIHC
TCGA.2Y.A9GS.01A	0.662034899	TCGA-LIHC
T724	0.660302937	CHCC-HBV
GSM363009	0.657835915	GSE14520
DO50791	0.657802467	LIRI-JP
TCGA.G3.A5SI.01A	0.657246525	TCGA-LIHC
TCGA.DD.AACB.01A	0.657136506	TCGA-LIHC
GSM1310591	0.656055552	GSE54236
GSM1310625	0.6552634	GSE54236
TCGA.WQ.A9G7.01A	0.65525193	TCGA-LIHC
GSM363204	0.654726503	GSE14520

GSM363331	0.654259447	GSE14520
GSM363182	0.653327332	GSE14520
TCGA.DD.AACG.01A	0.652875303	TCGA-LIHC
T573	0.652477067	CHCC-HBV
DO48706	0.651114352	LIRI-JP
GSM363052	0.650533162	GSE14520
TCGA.UB.A7MA.01A	0.649761612	TCGA-LIHC
DO23508	0.649680468	LIRI-JP
TCGA.CC.A8HV.01A	0.649272433	TCGA-LIHC
TCGA.DD.AACW.01A	0.649199819	TCGA-LIHC
GSM1310596	0.647517379	GSE54236
GSM363037	0.647256373	GSE14520
DO45173	0.647173481	LIRI-JP
GSM363100	0.647130048	GSE14520
DO23545	0.646895523	LIRI-JP
GSM363166	0.646783869	GSE14520
TCGA.2Y.A9H7.01A	0.646770954	TCGA-LIHC
GSM363106	0.646469243	GSE14520
DO48682	0.645486955	LIRI-JP
GSM363249	0.645485412	GSE14520
GSM363151	0.64473201	GSE14520
DO45301	0.644377301	LIRI-JP
TCGA.ED.A8O6.01A	0.644372209	TCGA-LIHC
GSM363013	0.644193493	GSE14520
TCGA.CC.A7IK.01A	0.644119804	TCGA-LIHC
T355	0.64354555	CHCC-HBV
TCGA.CC.A5UE.01A	0.643069917	TCGA-LIHC
T227	0.642718756	CHCC-HBV
GSM363017	0.642431278	GSE14520
DO45187	0.642274603	LIRI-JP
T131	0.641561171	CHCC-HBV
DO48723	0.641045246	LIRI-JP
T1013	0.640530286	CHCC-HBV
GSM363190	0.639779521	GSE14520
TCGA.QA.A7B7.01A	0.638930606	TCGA-LIHC
TCGA.CC.A7IJ.01A	0.638698975	TCGA-LIHC
TCGA.CC.A9FW.01A	0.637119187	TCGA-LIHC
GSM363271	0.636932181	GSE14520
TCGA.DD.A1EG.01A	0.636881785	TCGA-LIHC
T277	0.636798144	CHCC-HBV

DO48728	0.636787096	LIRI-JP
TCGA.G3.AAV5.01A	0.635364535	TCGA-LIHC
T313	0.635277415	CHCC-HBV
DO45205	0.634856	LIRI-JP
DO50809	0.634616887	LIRI-JP
GSM363348	0.634033412	GSE14520
DO45139	0.633593615	LIRI-JP
GSM363101	0.633534356	GSE14520
GSM363317	0.633286814	GSE14520
TCGA.MI.A75I.01A	0.632835318	TCGA-LIHC
GSM1310646	0.63230992	GSE54236
DO45245	0.63209655	LIRI-JP
TCGA.DD.AAD6.01A	0.630544809	TCGA-LIHC
TCGA.G3.A25Y.01A	0.629826829	TCGA-LIHC
DO48715	0.62927524	LIRI-JP
TCGA.DD.AAVU.01A	0.628842801	TCGA-LIHC
DO45117	0.62877997	LIRI-JP
TCGA.DD.AADR.01A	0.628718099	TCGA-LIHC
DO45277	0.627891465	LIRI-JP
GSM363049	0.627575638	GSE14520
GSM363079	0.626463835	GSE14520
TCGA.DD.AADO.01A	0.626273958	TCGA-LIHC
T375	0.625865054	CHCC-HBV
T525	0.625683962	CHCC-HBV
GSM1310650	0.625616297	GSE54236
T943	0.625538874	CHCC-HBV
TCGA.DD.A4NR.01A	0.625343947	TCGA-LIHC
TCGA.CC.5260.01A	0.625159776	TCGA-LIHC
GSM363339	0.624179103	GSE14520
GSM362993	0.623176189	GSE14520
T647	0.622201023	CHCC-HBV
TCGA.EP.A2KB.01A	0.62203243	TCGA-LIHC
TCGA.DD.AADF.01A	0.621932877	TCGA-LIHC
TCGA.G3.AAV7.01A	0.621677835	TCGA-LIHC
GSM363360	0.621574898	GSE14520
DO45239	0.621393529	LIRI-JP
TCGA.UB.A7ME.01A	0.621245406	TCGA-LIHC
GSM1310600	0.621216703	GSE54236
GSM1310648	0.621187527	GSE54236
T737	0.620300604	CHCC-HBV

GSM363069	0.620137447	GSE14520
DO23552	0.618008368	LIRI-JP
TCGA.BC.A217.01A	0.617755837	TCGA-LIHC
GSM363145	0.617743338	GSE14520
GSM363274	0.616666482	GSE14520
DO48732	0.616067145	LIRI-JP
GSM1310584	0.615876469	GSE54236
TCGA.ZP.A9D2.01A	0.615856738	TCGA-LIHC
TCGA.ED.A66X.01A	0.614645631	TCGA-LIHC
DO45219	0.61407782	LIRI-JP
GSM363174	0.613951236	GSE14520
GSM363170	0.613870603	GSE14520
T161	0.61345366	CHCC-HBV
T1021	0.613317947	CHCC-HBV
TCGA.DD.AAVS.01A	0.612931939	TCGA-LIHC
TCGA.BC.A10Y.01A	0.612778249	TCGA-LIHC
TCGA.DD.AADV.01A	0.612255191	TCGA-LIHC
DO45217	0.612207813	LIRI-JP
GSM363211	0.611661891	GSE14520
TCGA.BC.A8YO.01A	0.611096188	TCGA-LIHC
GSM363146	0.610957752	GSE14520
GSM1310635	0.610923332	GSE54236
TCGA.DD.AAEI.01A	0.610787179	TCGA-LIHC
TCGA.DD.AACV.01A	0.610576944	TCGA-LIHC
TCGA.BC.A10W.01A	0.610221032	TCGA-LIHC
GSM362986	0.610190643	GSE14520
T493	0.609614595	CHCC-HBV
DO45297	0.609272513	LIRI-JP
GSM1310585	0.608901955	GSE54236
T357	0.608206066	CHCC-HBV
T427	0.608090289	CHCC-HBV
DO48737	0.607591616	LIRI-JP
TCGA.CC.A3MA.01A	0.606198889	TCGA-LIHC
TCGA.ED.A66Y.01A	0.606158893	TCGA-LIHC
GSM363298	0.606007201	GSE14520
TCGA.DD.AACH.01A	0.604969755	TCGA-LIHC
T967	0.604103223	CHCC-HBV
GSM363326	0.604019657	GSE14520
GSM363192	0.603960792	GSE14520
TCGA.DD.AADM.01A	0.603727189	TCGA-LIHC

T257	0.603616946	CHCC-HBV
TCGA.DD.AAEE.01A	0.603127894	TCGA-LIHC
T491	0.602740007	CHCC-HBV
T883	0.602555152	CHCC-HBV
DO45225	0.602186591	LIRI-JP
T433	0.601775849	CHCC-HBV
DO45281	0.601624306	LIRI-JP
TCGA.2Y.A9H2.01A	0.601613523	TCGA-LIHC
T865	0.601220993	CHCC-HBV
TCGA.ED.A82E.01A	0.600868967	TCGA-LIHC
TCGA.EP.A2KA.01A	0.600667167	TCGA-LIHC
DO48747	0.600564553	LIRI-JP
DO45165	0.600057931	LIRI-JP
TCGA.K7.A5RG.01A	0.599546462	TCGA-LIHC
TCGA.CC.5264.01A	0.59865403	TCGA-LIHC
TCGA.UB.A7MF.01A	0.5986135	TCGA-LIHC
GSM1310643	0.598048104	GSE54236
TCGA.UB.A7MB.01A	0.597780361	TCGA-LIHC
T217	0.597666172	CHCC-HBV
TCGA.DD.A73F.01A	0.59766132	TCGA-LIHC
GSM363105	0.597386493	GSE14520
GSM363224	0.597131357	GSE14520
DO45237	0.596925237	LIRI-JP
DO45283	0.596436537	LIRI-JP
DO23524	0.596141724	LIRI-JP
TCGA.DD.AAC8.01A	0.595858399	TCGA-LIHC
GSM363056	0.595370726	GSE14520
GSM363329	0.595251008	GSE14520
GSM1310610	0.595077754	GSE54236
TCGA.RC.A7S9.01A	0.594866013	TCGA-LIHC
DO45183	0.594787603	LIRI-JP
GSM363311	0.594782879	GSE14520
GSM1310572	0.594766468	GSE54236
T953	0.594732866	CHCC-HBV
TCGA.UB.A7MC.01A	0.594047671	TCGA-LIHC
T545	0.593228259	CHCC-HBV
DO50840	0.592425613	LIRI-JP
TCGA.EP.A3RK.01A	0.592287475	TCGA-LIHC
GSM363232	0.592198285	GSE14520
GSM1310590	0.591570746	GSE54236

GSM363048	0.591402652	GSE14520
TCGA.DD.A4NQ.01A	0.59079452	TCGA-LIHC
DO48741	0.590545722	LIRI-JP
TCGA.G3.A25S.01A	0.590194643	TCGA-LIHC
TCGA.DD.AAD5.01A	0.589993146	TCGA-LIHC
T473	0.589777289	CHCC-HBV
T571	0.589684122	CHCC-HBV
DO48717	0.589493656	LIRI-JP
TCGA.2Y.A9H8.01A	0.589120726	TCGA-LIHC
GSM363314	0.589071258	GSE14520
GSM363198	0.589050968	GSE14520
TCGA.DD.AAVQ.01A	0.588356869	TCGA-LIHC
TCGA.DD.AACF.01A	0.588296044	TCGA-LIHC
TCGA.EP.A3JL.01A	0.588132571	TCGA-LIHC
TCGA.BC.A10Q.01A	0.587077821	TCGA-LIHC
TCGA.DD.AAE6.01A	0.5865189	TCGA-LIHC
TCGA.DD.AACK.01A	0.58645791	TCGA-LIHC
DO45179	0.586357886	LIRI-JP
T363	0.586311914	CHCC-HBV
GSM363270	0.586256026	GSE14520
DO48720	0.586242086	LIRI-JP
TCGA.BW.A5NO.01A	0.586084492	TCGA-LIHC
TCGA.BC.4073.01B	0.585044885	TCGA-LIHC
TCGA.G3.A5SJ.01A	0.584896323	TCGA-LIHC
TCGA.WX.AA44.01A	0.584565023	TCGA-LIHC
T817	0.584479384	CHCC-HBV
GSM363292	0.584426663	GSE14520
GSM363080	0.584197428	GSE14520
T663	0.58417611	CHCC-HBV
GSM1310620	0.583708265	GSE54236
GSM363086	0.582843696	GSE14520
DO45169	0.582542245	LIRI-JP
TCGA.DD.A39Z.01A	0.582481407	TCGA-LIHC
GSM362982	0.582256632	GSE14520
T665	0.581906377	CHCC-HBV
DO45257	0.581905796	LIRI-JP
TCGA.FV.A3I1.01A	0.581862218	TCGA-LIHC
DO45235	0.581148634	LIRI-JP
TCGA.DD.AADL.01A	0.580999611	TCGA-LIHC
T435	0.580910287	CHCC-HBV

TCGA.CC.5262.01A	0.580472495	TCGA-LIHC
GSM363072	0.580429358	GSE14520
DO45123	0.579720806	LIRI-JP
T517	0.579692122	CHCC-HBV
GSM1310601	0.579654743	GSE54236
TCGA.5C.AAPD.01A	0.579440389	TCGA-LIHC
DO45131	0.578027104	LIRI-JP
GSM363038	0.577960005	GSE14520
TCGA.DD.A113.01A	0.577910931	TCGA-LIHC
TCGA.2Y.A9HA.01A	0.577831181	TCGA-LIHC
DO23510	0.576719096	LIRI-JP
DO45213	0.576664446	LIRI-JP
TCGA.K7.AAU7.01A	0.576636874	TCGA-LIHC
TCGA.FV.A23B.01A	0.576483688	TCGA-LIHC
TCGA.DD.A4NE.01A	0.575812192	TCGA-LIHC
TCGA.2Y.A9H4.01A	0.575449319	TCGA-LIHC
TCGA.DD.A73B.01A	0.575302497	TCGA-LIHC
T917	0.575185668	CHCC-HBV
TCGA.G3.AAV1.01A	0.57500162	TCGA-LIHC
TCGA.PD.A5DF.01A	0.574652795	TCGA-LIHC
GSM363328	0.574462948	GSE14520
DO50802	0.574357984	LIRI-JP
TCGA.DD.AAEA.01A	0.573895496	TCGA-LIHC
TCGA.DD.AACX.01A	0.573706619	TCGA-LIHC
GSM363355	0.573555071	GSE14520
TCGA.G3.A25X.01A	0.573529468	TCGA-LIHC
TCGA.DD.A114.01A	0.573411614	TCGA-LIHC
T533	0.573408329	CHCC-HBV
GSM1310619	0.572641688	GSE54236
TCGA.RC.A7SH.01A	0.572638149	TCGA-LIHC
DO45199	0.571873068	LIRI-JP
DO50820	0.571218215	LIRI-JP
DO45119	0.570754918	LIRI-JP
GSM363400	0.570708702	GSE14520
TCGA.DD.AACJ.01A	0.570546874	TCGA-LIHC
DO50798	0.570477299	LIRI-JP
DO48679	0.569932064	LIRI-JP
TCGA.FV.A2QQ.01A	0.569731406	TCGA-LIHC
TCGA.DD.A3A3.01A	0.569717816	TCGA-LIHC
GSM363362	0.569608388	GSE14520

GSM362992	0.56931101	GSE14520
TCGA.BC.A10Z.01A	0.569244813	TCGA-LIHC
T957	0.569125891	CHCC-HBV
TCGA.ED.A8O5.01A	0.567951319	TCGA-LIHC
DO45092	0.56725701	LIRI-JP
DO45247	0.567211321	LIRI-JP
DO23549	0.567164034	LIRI-JP
TCGA.DD.AADQ.01A	0.567118322	TCGA-LIHC
TCGA.DD.A3A7.01A	0.56685262	TCGA-LIHC
GSM363215	0.566425581	GSE14520
DO50825	0.566361073	LIRI-JP
GSM363149	0.565479296	GSE14520
TCGA.DD.AAVZ.01A	0.564955958	TCGA-LIHC
GSM363266	0.564939951	GSE14520
TCGA.WX.AA47.01A	0.564627917	TCGA-LIHC
TCGA.DD.AAE1.01A	0.564513189	TCGA-LIHC
GSM363341	0.564056367	GSE14520
TCGA.G3.A7M7.01A	0.563841916	TCGA-LIHC
GSM363310	0.563792107	GSE14520
TCGA.DD.A4NJ.01A	0.563120521	TCGA-LIHC
DO45155	0.56305779	LIRI-JP
GSM363251	0.562398796	GSE14520
DO45163	0.562323164	LIRI-JP
DO23542	0.561739221	LIRI-JP
DO45096	0.561419671	LIRI-JP
TCGA.DD.A39V.01A	0.560808211	TCGA-LIHC
DO23528	0.560499422	LIRI-JP
TCGA.BC.A216.01A	0.56049403	TCGA-LIHC
TCGA.YA.A8S7.01A	0.56049296	TCGA-LIHC
DO48736	0.560169194	LIRI-JP
TCGA.DD.AADJ.01A	0.559694727	TCGA-LIHC
TCGA.DD.AACA.01A	0.559690037	TCGA-LIHC
GSM363172	0.559174108	GSE14520
TCGA.DD.A1EH.01A	0.558595675	TCGA-LIHC
DO48712	0.558562063	LIRI-JP
TCGA.XR.A8TF.01A	0.558246752	TCGA-LIHC
DO45231	0.558191584	LIRI-JP
GSM362959	0.557958124	GSE14520
TCGA.CC.5259.01A	0.557953205	TCGA-LIHC
T135	0.55759227	CHCC-HBV

GSM363152	0.557230797	GSE14520
T127	0.557034165	CHCC-HBV
DO23516	0.557001811	LIRI-JP
GSM362970	0.55685207	GSE14520
TCGA.XR.A8TC.01A	0.556586554	TCGA-LIHC
T123	0.556360404	CHCC-HBV
GSM1310622	0.555857274	GSE54236
T685	0.55531575	CHCC-HBV
GSM363078	0.555060939	GSE14520
TCGA.CC.A3MC.01A	0.554839255	TCGA-LIHC
TCGA.5C.A9VH.01A	0.554718876	TCGA-LIHC
T267	0.554167975	CHCC-HBV
GSM363036	0.553646018	GSE14520
TCGA.DD.A1EI.01A	0.553296124	TCGA-LIHC
TCGA.BD.A2L6.01A	0.553263084	TCGA-LIHC
GSM1310644	0.552785477	GSE54236
GSM1310639	0.551843548	GSE54236
TCGA.MI.A75C.01A	0.55144391	TCGA-LIHC
DO45263	0.551378399	LIRI-JP
GSM363209	0.550576015	GSE14520
DO45253	0.550539336	LIRI-JP
TCGA.G3.AAV3.01A	0.550305785	TCGA-LIHC
TCGA.ED.A7XP.01A	0.550260134	TCGA-LIHC
T467	0.549974449	CHCC-HBV
TCGA.DD.AACC.01A	0.549631709	TCGA-LIHC
DO50822	0.549381523	LIRI-JP
DO23533	0.54929294	LIRI-JP
TCGA.RC.A6M4.01A	0.549027586	TCGA-LIHC
DO50811	0.54871171	LIRI-JP
T425	0.548520755	CHCC-HBV
TCGA.CC.A9FS.01A	0.548272524	TCGA-LIHC
TCGA.DD.AADP.01A	0.54803889	TCGA-LIHC
DO48725	0.548010929	LIRI-JP
TCGA.DD.A73G.01A	0.54796332	TCGA-LIHC
DO50844	0.54735934	LIRI-JP
GSM363125	0.547205567	GSE14520
GSM363050	0.546798738	GSE14520
GSM1310574	0.546571715	GSE54236
TCGA.DD.AACS.01A	0.546486776	TCGA-LIHC
TCGA.EP.A2KC.01A	0.545952117	TCGA-LIHC

TCGA.BC.A3KF.01A	0.545927794	TCGA-LIHC
GSM363247	0.545192249	GSE14520
GSM362958	0.545117308	GSE14520
TCGA.XR.A8TD.01A	0.545112651	TCGA-LIHC
DO45157	0.544994193	LIRI-JP
TCGA.G3.A25U.01A	0.544739469	TCGA-LIHC
DO23514	0.544590234	LIRI-JP
TCGA.DD.A3A1.01A	0.544338414	TCGA-LIHC
DO45091	0.544032382	LIRI-JP
TCGA.DD.AAD2.01A	0.543956967	TCGA-LIHC
DO45115	0.543842303	LIRI-JP
GSM363388	0.542506384	GSE14520
GSM1310611	0.542135248	GSE54236
TCGA.ED.A459.01A	0.542053932	TCGA-LIHC
TCGA.DD.A4NH.01A	0.541748246	TCGA-LIHC
TCGA.RC.A7SK.01A	0.541368879	TCGA-LIHC
GSM363031	0.54134128	GSE14520
DO48727	0.54107469	LIRI-JP
TCGA.DD.A73A.01A	0.541064409	TCGA-LIHC
GSM1310594	0.540696608	GSE54236
TCGA.RC.A7SF.01A	0.540544221	TCGA-LIHC
TCGA.DD.A3A9.01A	0.54041731	TCGA-LIHC
GSM1310624	0.540251391	GSE54236
TCGA.DD.A118.01A	0.54022521	TCGA-LIHC
TCGA.G3.A25Z.01A	0.540086154	TCGA-LIHC
TCGA.CC.5261.01A	0.539610643	TCGA-LIHC
GSM363123	0.539443974	GSE14520
TCGA.DD.AADI.01A	0.539102952	TCGA-LIHC
TCGA.DD.A73D.01A	0.538692719	TCGA-LIHC
TCGA.2Y.A9H9.01A	0.538580388	TCGA-LIHC
DO45289	0.538373247	LIRI-JP
TCGA.T1.A6J8.01A	0.537923911	TCGA-LIHC
DO50832	0.537916166	LIRI-JP
TCGA.BC.4072.01B	0.537651241	TCGA-LIHC
GSM1310627	0.537602257	GSE54236
TCGA.ED.A7XO.01A	0.537094605	TCGA-LIHC
DO48704	0.536270297	LIRI-JP
GSM363109	0.536014525	GSE14520
TCGA.DD.AAD0.01A	0.535902422	TCGA-LIHC
TCGA.CC.A7IF.01A	0.535900305	TCGA-LIHC

DO23543	0.535817628	LIRI-JP
DO45121	0.534175949	LIRI-JP
T527	0.534080038	CHCC-HBV
GSM1310632	0.533859424	GSE54236
TCGA.RC.A7SB.01A	0.53365944	TCGA-LIHC
TCGA.BC.A5W4.01A	0.533655061	TCGA-LIHC
GSM363053	0.533515987	GSE14520
T553	0.533369904	CHCC-HBV
DO50804	0.533342606	LIRI-JP
GSM1310575	0.533199186	GSE54236
TCGA.DD.A3A5.01A	0.533096572	TCGA-LIHC
TCGA.DD.AAW3.01A	0.53300387	TCGA-LIHC
GSM363269	0.532630149	GSE14520
TCGA.G3.A7M6.01A	0.53259992	TCGA-LIHC
DO50819	0.532239267	LIRI-JP
TCGA.KR.A7K2.01A	0.532199169	TCGA-LIHC
TCGA.O8.A75V.01A	0.53216355	TCGA-LIHC
GSM363169	0.531135443	GSE14520
DO50859	0.531031691	LIRI-JP
GSM363289	0.53045961	GSE14520
TCGA.CC.A7IL.01A	0.529773528	TCGA-LIHC
GSM1310621	0.529423268	GSE54236
GSM1310570	0.528554742	GSE54236
DO23509	0.528529638	LIRI-JP
TCGA.FV.A496.01A	0.528386324	TCGA-LIHC
TCGA.2Y.A9H5.01A	0.528022635	TCGA-LIHC
T635	0.527603519	CHCC-HBV
T741	0.527509242	CHCC-HBV
TCGA.DD.AADK.01A	0.526875015	TCGA-LIHC
TCGA.DD.A4NG.01A	0.526862008	TCGA-LIHC
TCGA.BD.A3EP.01A	0.526853305	TCGA-LIHC
TCGA.DD.A4NA.01A	0.526527732	TCGA-LIHC
DO23539	0.526022222	LIRI-JP
T125	0.52599033	CHCC-HBV
T221	0.525714266	CHCC-HBV
TCGA.ED.A7PY.01A	0.525589276	TCGA-LIHC
TCGA.FV.A3I0.01A	0.525357371	TCGA-LIHC
TCGA.DD.A115.01A	0.525300477	TCGA-LIHC
TCGA.DD.A39X.01A	0.525282163	TCGA-LIHC
T727	0.525201369	CHCC-HBV

TCGA.FV.A3R2.01A	0.524962191	TCGA-LIHC
GSM363148	0.524596372	GSE14520
GSM363207	0.524376883	GSE14520
TCGA.ED.A5KG.01A	0.523730878	TCGA-LIHC
TCGA.DD.AACD.01A	0.523634062	TCGA-LIHC
GSM1310580	0.522982221	GSE54236
TCGA.DD.A119.01A	0.522614441	TCGA-LIHC
TCGA.DD.A11C.01A	0.522252854	TCGA-LIHC
TCGA.DD.A11A.01A	0.521921994	TCGA-LIHC
DO48695	0.521722364	LIRI-JP
TCGA.UB.AA0U.01A	0.521601638	TCGA-LIHC
DO45143	0.521509719	LIRI-JP
TCGA.BC.A10T.01A	0.52119395	TCGA-LIHC
GSM363368	0.519540685	GSE14520
TCGA.DD.A39W.01A	0.51946583	TCGA-LIHC
DO23523	0.519336027	LIRI-JP
GSM363144	0.518694327	GSE14520
TCGA.BC.A10U.01A	0.518215423	TCGA-LIHC
DO45285	0.518131455	LIRI-JP
TCGA.2Y.A9GU.01A	0.517944286	TCGA-LIHC
T393	0.517295504	CHCC-HBV
TCGA.3K.AAZ8.01A	0.51722707	TCGA-LIHC
T431	0.516379196	CHCC-HBV
DO45175	0.516007603	LIRI-JP
TCGA.DD.AACA.02A	0.515850829	TCGA-LIHC
GSM363239	0.515776171	GSE14520
T1043	0.515733485	CHCC-HBV
GSM363230	0.515497763	GSE14520
DO45273	0.515307337	LIRI-JP
GSM1310588	0.514900334	GSE54236
TCGA.FV.A4ZP.01A	0.514874623	TCGA-LIHC
TCGA.ZS.A9CF.02A	0.514797232	TCGA-LIHC
DO45135	0.514739661	LIRI-JP
GSM1310647	0.513918068	GSE54236
TCGA.DD.AAE4.01A	0.513685138	TCGA-LIHC
GSM1310649	0.513386786	GSE54236
TCGA.DD.AACO.01A	0.513110585	TCGA-LIHC
DO50857	0.512550035	LIRI-JP
T1031	0.512469389	CHCC-HBV
GSM1310637	0.512455263	GSE54236

GSM1310645	0.511822802	GSE54236
TCGA.DD.AAW2.01A	0.511726418	TCGA-LIHC
T487	0.511101844	CHCC-HBV
DO23550	0.51090154	LIRI-JP
TCGA.FV.A495.01A	0.510871292	TCGA-LIHC
T171	0.510832385	CHCC-HBV
T443	0.510604506	CHCC-HBV
GSM363241	0.510452141	GSE14520
GSM363057	0.509942019	GSE14520
GSM1310616	0.509845865	GSE54236
DO45137	0.509401313	LIRI-JP
T755	0.509333198	CHCC-HBV
DO50855	0.509175955	LIRI-JP
DO50850	0.508951567	LIRI-JP
GSM1310595	0.508428761	GSE54236
TCGA.DD.AAC9.01A	0.508267033	TCGA-LIHC
DO45255	0.508185766	LIRI-JP
T713	0.507216285	CHCC-HBV
TCGA.MI.A75G.01A	0.50721347	TCGA-LIHC
GSM363107	0.507186916	GSE14520
TCGA.GJ.A9DB.01A	0.507097161	TCGA-LIHC
TCGA.2Y.A9H1.01A	0.507062506	TCGA-LIHC
GSM363180	0.5069334	GSE14520
GSM363336	0.506871727	GSE14520
TCGA.DD.AAE9.01A	0.506687641	TCGA-LIHC
DO45099	0.506637746	LIRI-JP
GSM363075	0.506524898	GSE14520
T523	0.506461239	CHCC-HBV
T975	0.506005974	CHCC-HBV
GSM363265	0.505641062	GSE14520
DO45203	0.505460443	LIRI-JP
TCGA.DD.A1EC.01A	0.50541343	TCGA-LIHC
T141	0.504939835	CHCC-HBV
GSM1310608	0.504928661	GSE54236
GSM1310617	0.50488664	GSE54236
TCGA.MI.A75H.01A	0.504788045	TCGA-LIHC
GSM1310602	0.504642961	GSE54236
T1015	0.50460328	CHCC-HBV
DO48701	0.504524423	LIRI-JP
TCGA.GJ.A6C0.01A	0.504460344	TCGA-LIHC

T361	0.504420832	CHCC-HBV
TCGA.DD.AACQ.01A	0.50432613	TCGA-LIHC
TCGA.DD.AACU.01A	0.504173874	TCGA-LIHC
GSM363178	0.503670409	GSE14520
T455	0.503497912	CHCC-HBV
DO50818	0.50345299	LIRI-JP
T311	0.503215586	CHCC-HBV
DO45113	0.502617864	LIRI-JP
GSM363346	0.502565598	GSE14520
TCGA.DD.AAE7.01A	0.502553587	TCGA-LIHC
TCGA.DD.AAD8.01A	0.502506697	TCGA-LIHC
TCGA.WQ.AB4B.01A	0.502342584	TCGA-LIHC
TCGA.EP.A26S.01A	0.502029873	TCGA-LIHC
GSM363082	0.50189495	GSE14520
DO23546	0.500902687	LIRI-JP
TCGA.MI.A75E.01A	0.500643761	TCGA-LIHC
T1025	0.500622971	CHCC-HBV
TCGA.ZP.A9D4.01A	0.500561809	TCGA-LIHC
GSM1310597	0.500515538	GSE54236
GSM363071	0.500045601	GSE14520
T923	0.499874936	CHCC-HBV
GSM1310623	0.499852126	GSE54236
GSM363337	0.499832607	GSE14520
DO45153	0.499824565	LIRI-JP
TCGA.G3.AAUZ.01A	0.49979771	TCGA-LIHC
TCGA.G3.A5SL.01A	0.499712031	TCGA-LIHC
DO50803	0.499344749	LIRI-JP
GSM363315	0.499197953	GSE14520
GSM363391	0.498653052	GSE14520
GSM363291	0.49858949	GSE14520
GSM1310609	0.498550275	GSE54236
DO45097	0.498433921	LIRI-JP
GSM1310605	0.498143044	GSE54236
TCGA.DD.A116.01A	0.497699566	TCGA-LIHC
GSM1310630	0.497364302	GSE54236
TCGA.2Y.A9GW.01A	0.496852973	TCGA-LIHC
TCGA.XR.A8TG.01A	0.49598908	TCGA-LIHC
T231	0.4955588	CHCC-HBV
TCGA.G3.AAV4.01A	0.495447062	TCGA-LIHC
TCGA.DD.A1EE.01A	0.495206876	TCGA-LIHC

DO23541	0.495155019	LIRI-JP
DO45191	0.494113542	LIRI-JP
GSM1310593	0.49410094	GSE54236
DO23536	0.494046831	LIRI-JP
GSM363220	0.493656385	GSE14520
T113	0.493624498	CHCC-HBV
GSM363205	0.493517249	GSE14520
GSM362966	0.493422972	GSE14520
GSM363217	0.493158002	GSE14520
TCGA.DD.AAVV.01A	0.49302644	TCGA-LIHC
GSM1310592	0.492526242	GSE54236
DO50793	0.492424156	LIRI-JP
DO48761	0.492382991	LIRI-JP
DO48759	0.4920722	LIRI-JP
GSM363358	0.49192526	GSE14520
T483	0.491472465	CHCC-HBV
TCGA.BD.A3ER.01A	0.491467252	TCGA-LIHC
DO45227	0.4914639	LIRI-JP
DO50805	0.490535663	LIRI-JP
DO50807	0.490220241	LIRI-JP
TCGA.2Y.A9HB.01A	0.490192554	TCGA-LIHC
GSM363290	0.489917296	GSE14520
DO50776	0.489542939	LIRI-JP
DO50808	0.489069365	LIRI-JP
GSM363213	0.488070445	GSE14520
GSM363294	0.487837504	GSE14520
TCGA.DD.AAVY.01A	0.487513335	TCGA-LIHC
GSM1310589	0.487479851	GSE54236
TCGA.DD.A3A4.01A	0.486983488	TCGA-LIHC
TCGA.5R.AA1C.01A	0.486980839	TCGA-LIHC
T471	0.486588272	CHCC-HBV
GSM363202	0.486212032	GSE14520
T147	0.485466426	CHCC-HBV
TCGA.G3.AAV2.01A	0.485187879	TCGA-LIHC
DO50814	0.484652707	LIRI-JP
T112	0.484132194	CHCC-HBV
T187	0.483837693	CHCC-HBV
TCGA.G3.A3CK.01A	0.483053593	TCGA-LIHC
TCGA.DD.AAVR.01A	0.48299733	TCGA-LIHC
DO45171	0.482599015	LIRI-JP

GSM363128	0.482474803	GSE14520
TCGA.G3.A5SM.01A	0.482050041	TCGA-LIHC
TCGA.DD.AACN.01A	0.48183518	TCGA-LIHC
GSM363376	0.481700441	GSE14520
GSM1310615	0.481524145	GSE54236
TCGA.DD.AACT.01A	0.481227936	TCGA-LIHC
TCGA.DD.AAVX.01A	0.481212312	TCGA-LIHC
DO48700	0.480343817	LIRI-JP
TCGA.4R.AA8I.01A	0.479935973	TCGA-LIHC
GSM712542	0.479539636	GSE14520
T383	0.479502421	CHCC-HBV
TCGA.GJ.A3OU.01A	0.479030585	TCGA-LIHC
TCGA.DD.AADY.01A	0.478507142	TCGA-LIHC
DO48716	0.478445654	LIRI-JP
TCGA.DD.AACY.01A	0.478439097	TCGA-LIHC
TCGA.DD.AAEH.01A	0.478076512	TCGA-LIHC
TCGA.K7.A6G5.01A	0.477850818	TCGA-LIHC
T271	0.477824561	CHCC-HBV
GSM1310571	0.477821312	GSE54236
GSM363015	0.477432995	GSE14520
DO23529	0.477255233	LIRI-JP
TCGA.DD.AAVP.01A	0.477223888	TCGA-LIHC
DO23518	0.477106045	LIRI-JP
DO48743	0.476670538	LIRI-JP
TCGA.ES.A2HS.01A	0.476385438	TCGA-LIHC
GSM1310606	0.476249352	GSE54236
GSM1310631	0.475376104	GSE54236
TCGA.DD.AADG.01A	0.475133276	TCGA-LIHC
GSM362987	0.474975394	GSE14520
TCGA.G3.A3CJ.01A	0.474930874	TCGA-LIHC
TCGA.G3.A7M5.01A	0.474869499	TCGA-LIHC
TCGA.2Y.A9H3.01A	0.474730257	TCGA-LIHC
GSM363343	0.473530546	GSE14520
GSM363313	0.473146434	GSE14520
DO45249	0.472423196	LIRI-JP
GSM1310613	0.472071012	GSE54236
T921	0.471864862	CHCC-HBV
TCGA.UB.A7MD.01A	0.471859588	TCGA-LIHC
TCGA.G3.A3CH.01A	0.471666669	TCGA-LIHC
GSM1310583	0.471658668	GSE54236

TCGA.DD.AACI.01A	0.471539966	TCGA-LIHC
T851	0.471351183	CHCC-HBV
T223	0.470928335	CHCC-HBV
T477	0.469917655	CHCC-HBV
DO23540	0.469811234	LIRI-JP
TCGA.CC.A7IH.01A	0.46953002	TCGA-LIHC
GSM363164	0.469527557	GSE14520
GSM363115	0.469240255	GSE14520
GSM363333	0.469121631	GSE14520
DO50783	0.469061345	LIRI-JP
GSM362960	0.468950761	GSE14520
GSM1310604	0.468231916	GSE54236
T695	0.468184679	CHCC-HBV
T465	0.467523464	CHCC-HBV
DO45197	0.467376308	LIRI-JP
DO48672	0.467314935	LIRI-JP
TCGA.ZS.A9CE.01A	0.467288829	TCGA-LIHC
TCGA.DD.AACE.01A	0.467224053	TCGA-LIHC
DO50813	0.467007533	LIRI-JP
GSM1310636	0.466757899	GSE54236
TCGA.DD.AAE3.01A	0.466270257	TCGA-LIHC
GSM1310598	0.46599675	GSE54236
TCGA.DD.A1EA.01A	0.465970774	TCGA-LIHC
TCGA.KR.A7K8.01A	0.46559267	TCGA-LIHC
TCGA.FV.A2QR.01A	0.464779793	TCGA-LIHC
T557	0.464422917	CHCC-HBV
T913	0.464140926	CHCC-HBV
TCGA.DD.AADU.01A	0.464122455	TCGA-LIHC
GSM1310634	0.464064807	GSE54236
TCGA.ZS.A9CD.01A	0.463770333	TCGA-LIHC
TCGA.DD.AAE2.01A	0.463598448	TCGA-LIHC
GSM362965	0.463327019	GSE14520
TCGA.DD.AAEG.01A	0.462468356	TCGA-LIHC
DO48693	0.462377946	LIRI-JP
TCGA.G3.A7M8.01A	0.462361066	TCGA-LIHC
DO45243	0.462228226	LIRI-JP
DO23513	0.461283689	LIRI-JP
T341	0.461205794	CHCC-HBV
GSM363267	0.461060701	GSE14520
TCGA.DD.A4NO.01A	0.460300427	TCGA-LIHC

T925	0.460268712	CHCC-HBV
GSM1310640	0.460092324	GSE54236
DO45095	0.460035111	LIRI-JP
TCGA.KR.A7K0.01A	0.459962144	TCGA-LIHC
TCGA.XR.A8TE.01A	0.45958449	TCGA-LIHC
TCGA.DD.A3A8.01A	0.459125365	TCGA-LIHC
DO45207	0.458565201	LIRI-JP
TCGA.NI.A4U2.01A	0.458063002	TCGA-LIHC
T641	0.457745974	CHCC-HBV
TCGA.DD.AAEK.01A	0.457563508	TCGA-LIHC
TCGA.DD.A73E.01A	0.457219409	TCGA-LIHC
GSM363014	0.456665934	GSE14520
TCGA.G3.AAV0.01A	0.456519037	TCGA-LIHC
TCGA.ZP.A9D0.01A	0.456442113	TCGA-LIHC
TCGA.LG.A6GG.01A	0.455781024	TCGA-LIHC
DO45251	0.455440501	LIRI-JP
GSM362988	0.454964072	GSE14520
DO227801	0.454716904	LIRI-JP
TCGA.G3.A3CG.01A	0.454689205	TCGA-LIHC
TCGA.MR.A520.01A	0.454271963	TCGA-LIHC
GSM1310638	0.454215373	GSE54236
TCGA.DD.AADA.01A	0.453063026	TCGA-LIHC
TCGA.2Y.A9H6.01A	0.453038918	TCGA-LIHC
GSM363130	0.452925508	GSE14520
TCGA.G3.A25V.01A	0.452443411	TCGA-LIHC
T745	0.452010439	CHCC-HBV
DO23535	0.452007751	LIRI-JP
TCGA.LG.A9QC.01A	0.451653477	TCGA-LIHC
TCGA.ZP.A9CV.01A	0.451104093	TCGA-LIHC
T823	0.451062409	CHCC-HBV
TCGA.BC.A10S.01A	0.451059917	TCGA-LIHC
GSM363371	0.450909946	GSE14520
TCGA.2Y.A9GZ.01A	0.450760195	TCGA-LIHC
GSM363352	0.450334787	GSE14520
T451	0.450107901	CHCC-HBV
TCGA.DD.A1EK.01A	0.449776569	TCGA-LIHC
TCGA.DD.AAED.01A	0.449415355	TCGA-LIHC
TCGA.DD.A3A2.01A	0.447255377	TCGA-LIHC
TCGA.ES.A2HT.01A	0.446618743	TCGA-LIHC
DO48677	0.446255788	LIRI-JP

GSM363188	0.444936227	GSE14520
T977	0.444924864	CHCC-HBV
TCGA.DD.AAD1.01A	0.444680317	TCGA-LIHC
TCGA.LG.A9QD.01A	0.444678288	TCGA-LIHC
GSM363168	0.444346168	GSE14520
TCGA.WJ.A86L.01A	0.444050406	TCGA-LIHC
TCGA.MR.A8JO.01A	0.4436944	TCGA-LIHC
GSM363404	0.443681494	GSE14520
DO50789	0.442315241	LIRI-JP
DO45229	0.441516104	LIRI-JP
GSM363008	0.441316038	GSE14520
GSM363288	0.441088025	GSE14520
GSM1310587	0.440805	GSE54236
T813	0.440613819	CHCC-HBV
T445	0.439026835	CHCC-HBV
T1027	0.438975258	CHCC-HBV
DO48760	0.438933128	LIRI-JP
T873	0.438658113	CHCC-HBV
TCGA.DD.AAVW.01A	0.437797504	TCGA-LIHC
TCGA.ZS.A9CF.01A	0.43736121	TCGA-LIHC
T421	0.437306968	CHCC-HBV
GSM363029	0.437034505	GSE14520
DO45133	0.436066589	LIRI-JP
GSM1310628	0.435601257	GSE54236
T937	0.435475157	CHCC-HBV
T461	0.435221687	CHCC-HBV
T415	0.434524685	CHCC-HBV
TCGA.DD.A4ND.01A	0.433026049	TCGA-LIHC
DO23511	0.432870519	LIRI-JP
T715	0.432824757	CHCC-HBV
GSM363226	0.432529429	GSE14520
TCGA.BC.A10R.01A	0.432162546	TCGA-LIHC
TCGA.DD.AADS.01A	0.431772016	TCGA-LIHC
TCGA.G3.A3CI.01A	0.431175444	TCGA-LIHC
DO45223	0.431007821	LIRI-JP
T861	0.430636933	CHCC-HBV
GSM1310641	0.429429358	GSE54236
DO50787	0.429304738	LIRI-JP
GSM362964	0.429087189	GSE14520
GSM1310618	0.428727168	GSE54236

DO45129	0.428501866	LIRI-JP
GSM1310607	0.428424452	GSE54236
TCGA.DD.A73C.01A	0.428213391	TCGA-LIHC
DO45103	0.428095735	LIRI-JP
GSM363034	0.427397196	GSE14520
TCGA.DD.AAD3.01A	0.427079525	TCGA-LIHC
GSM362978	0.427040245	GSE14520
GSM1310626	0.42680533	GSE54236
DO48721	0.426441275	LIRI-JP
T963	0.42484979	CHCC-HBV
GSM363196	0.424356824	GSE14520
TCGA.HP.A5MZ.01A	0.423215632	TCGA-LIHC
DO48742	0.422712586	LIRI-JP
TCGA.ZP.A9D1.01A	0.422528147	TCGA-LIHC
GSM363102	0.422321566	GSE14520
TCGA.BC.A69I.01A	0.422182136	TCGA-LIHC
GSM363344	0.420442781	GSE14520
DO23548	0.420257746	LIRI-JP
TCGA.ZP.A9CY.01A	0.420170735	TCGA-LIHC
GSM363108	0.420105691	GSE14520
DO48733	0.419816929	LIRI-JP
T365	0.418042063	CHCC-HBV
T881	0.417853988	CHCC-HBV
GSM363039	0.417003044	GSE14520
T195	0.416147851	CHCC-HBV
DO48719	0.41607737	LIRI-JP
DO48746	0.415681463	LIRI-JP
TCGA.DD.A11B.01A	0.415009746	TCGA-LIHC
T1045	0.414806874	CHCC-HBV
GSM363327	0.41436434	GSE14520
GSM363200	0.412423724	GSE14520
DO45105	0.412265188	LIRI-JP
DO23538	0.4119677	LIRI-JP
DO23547	0.411883185	LIRI-JP
DO45159	0.410823133	LIRI-JP
DO45177	0.410762835	LIRI-JP
T145	0.410074644	CHCC-HBV
TCGA.DD.AAW1.01A	0.409768497	TCGA-LIHC
DO227643	0.409487599	LIRI-JP
GSM363272	0.409274379	GSE14520

TCGA.DD.AAW0.01A	0.40792445	TCGA-LIHC
DO45261	0.407759656	LIRI-JP
GSM363293	0.406065477	GSE14520
TCGA.DD.A1EB.01A	0.405750712	TCGA-LIHC
T1041	0.405322744	CHCC-HBV
TCGA.DD.AAEB.01A	0.405261997	TCGA-LIHC
DO23527	0.40337034	LIRI-JP
TCGA.DD.A4NI.01A	0.402465297	TCGA-LIHC
T563	0.40224311	CHCC-HBV
TCGA.ED.A4XI.01A	0.399617632	TCGA-LIHC
GSM362994	0.399270937	GSE14520
T615	0.398516345	CHCC-HBV
DO45094	0.397219741	LIRI-JP
DO45141	0.396753439	LIRI-JP
DO50774	0.396717151	LIRI-JP
DO45149	0.396529018	LIRI-JP
DO45195	0.39616035	LIRI-JP
T777	0.396084502	CHCC-HBV
GSM363264	0.395995428	GSE14520
DO45233	0.395285793	LIRI-JP
GSM363084	0.394647544	GSE14520
TCGA.EP.A12J.01A	0.394478328	TCGA-LIHC
DO23512	0.393724723	LIRI-JP
TCGA.DD.A4NP.01A	0.393450056	TCGA-LIHC
T515	0.392857993	CHCC-HBV
DO45201	0.392434872	LIRI-JP
GSM363126	0.392024775	GSE14520
TCGA.NI.A8LF.01A	0.391743638	TCGA-LIHC
DO45241	0.391023809	LIRI-JP
T423	0.3881157	CHCC-HBV
TCGA.WX.AA46.01A	0.387663979	TCGA-LIHC
DO50839	0.387494635	LIRI-JP
GSM363012	0.386956067	GSE14520
TCGA.UB.AA0V.01A	0.386394287	TCGA-LIHC
TCGA.DD.A11D.01A	0.385783791	TCGA-LIHC
TCGA.5R.AAAM.01A	0.385025511	TCGA-LIHC
T513	0.383969046	CHCC-HBV
TCGA.G3.A6UC.01A	0.383775193	TCGA-LIHC
TCGA.DD.A4NK.01A	0.38279019	TCGA-LIHC
DO45181	0.380633543	LIRI-JP

GSM363081	0.380242919	GSE14520
TCGA.DD.A4NF.01A	0.379668955	TCGA-LIHC
T395	0.379197193	CHCC-HBV
DO48674	0.378679854	LIRI-JP
GSM363366	0.378548096	GSE14520
TCGA.2Y.A9GX.01A	0.376313623	TCGA-LIHC
TCGA.ZS.A9CG.01A	0.376177225	TCGA-LIHC
T617	0.374790641	CHCC-HBV
TCGA.ED.A97K.01A	0.374263987	TCGA-LIHC
GSM363054	0.370612593	GSE14520
T137	0.369733444	CHCC-HBV
TCGA.RC.A6M5.01A	0.369553203	TCGA-LIHC
GSM363312	0.36939872	GSE14520
T785	0.369103751	CHCC-HBV
DO23517	0.367014473	LIRI-JP
GSM363104	0.365118588	GSE14520
DO50800	0.362764039	LIRI-JP
DO50816	0.362636856	LIRI-JP
DO45125	0.362315855	LIRI-JP
GSM363273	0.361027013	GSE14520
GSM362972	0.358637899	GSE14520
TCGA.DD.A4NL.01A	0.358027874	TCGA-LIHC
TCGA.2Y.A9GV.01A	0.35774233	TCGA-LIHC
T863	0.357526387	CHCC-HBV
T911	0.354815933	CHCC-HBV
TCGA.2Y.A9GT.01A	0.353070659	TCGA-LIHC
TCGA.BC.A10X.01A	0.349699281	TCGA-LIHC
DO23531	0.349102222	LIRI-JP
T261	0.348267196	CHCC-HBV
TCGA.FV.A3R3.01A	0.34812573	TCGA-LIHC
T353	0.34807199	CHCC-HBV
GSM363087	0.347924811	GSE14520
GSM1310578	0.347894045	GSE54236
T327	0.345653278	CHCC-HBV
TCGA.DD.A4NV.01A	0.345154613	TCGA-LIHC
TCGA.HP.A5N0.01A	0.344455886	TCGA-LIHC
GSM363016	0.343937155	GSE14520
DO45111	0.343893689	LIRI-JP
T627	0.343552392	CHCC-HBV
DO23544	0.342074128	LIRI-JP

TCGA.BC.A110.01A	0.341714879	TCGA-LIHC
GSM363074	0.341312669	GSE14520
GSM363335	0.341086257	GSE14520
DO23534	0.341060702	LIRI-JP
TCGA.ED.A627.01A	0.340087852	TCGA-LIHC
TCGA.5R.AA1D.01A	0.339651893	TCGA-LIHC
GSM1310581	0.338327255	GSE54236
DO45279	0.337028358	LIRI-JP
GSM363176	0.336959042	GSE14520
DO23530	0.336158558	LIRI-JP
GSM363124	0.333265961	GSE14520
T867	0.333097039	CHCC-HBV
DO45167	0.331709788	LIRI-JP
GSM363099	0.331152402	GSE14520
DO45109	0.330850144	LIRI-JP
GSM363142	0.329003609	GSE14520
GSM1310582	0.32883125	GSE54236
T915	0.328829044	CHCC-HBV
TCGA.G3.A5SK.01A	0.328657082	TCGA-LIHC
DO50817	0.328462806	LIRI-JP
T481	0.327456509	CHCC-HBV
GSM363364	0.325799626	GSE14520
DO45101	0.325655674	LIRI-JP
GSM362976	0.325020396	GSE14520
GSM363295	0.32494396	GSE14520
DO50845	0.323560985	LIRI-JP
GSM363121	0.32259432	GSE14520
DO45107	0.32223099	LIRI-JP
GSM362984	0.320883677	GSE14520
TCGA.DD.A4NB.01A	0.319338534	TCGA-LIHC
GSM363186	0.318781467	GSE14520
TCGA.K7.A5RF.01A	0.318342364	TCGA-LIHC
T955	0.315598974	CHCC-HBV
TCGA.DD.A1ED.01A	0.313861651	TCGA-LIHC
DO23551	0.313630083	LIRI-JP
T191	0.311925892	CHCC-HBV
DO45215	0.308580704	LIRI-JP
GSM363055	0.304845609	GSE14520
T981	0.304701056	CHCC-HBV
TCGA.DD.A4NS.01A	0.301903014	TCGA-LIHC

T857	0.300210578	CHCC-HBV
GSM363010	0.278412038	GSE14520
GSM363378	0.269455066	GSE14520
GSM362977	0.268221341	GSE14520
GSM1310577	0.268006547	GSE54236
GSM363035	0.263813743	GSE14520
TCGA.DD.A3A6.01A	0.263610893	TCGA-LIHC
GSM363243	0.259939581	GSE14520
GSM363051	0.232938639	GSE14520
GSM363098	0.220697442	GSE14520
GSM363275	0.21999747	GSE14520
GSM363393	0.218179727	GSE14520
GSM362971	0.199558683	GSE14520
T721	0.19940148	CHCC-HBV
GSM363073	0.198429789	GSE14520
GSM1310629	0.197018103	GSE54236
GSM363122	0.186528516	GSE14520
DO23532	0.177896555	LIRI-JP
GSM363143	0.170065957	GSE14520
GSM1310579	0.055940101	GSE54236
GSM363076	0	GSE14520

Table S3. Thirteen HSRM hub genes selected by LASSO regression models.

Gene Symbol	HSRM coefficient	HR[a]	HR.95L[a]	HR.95H[a]	P-value[a]
KPNA2	-0.0426	1.758	1.454	2.125	5.692E-09
UCK2	0.1762	1.960	1.553	2.475	1.469E-08
PPM1G	-0.0460	2.462	1.789	3.390	3.295E-08
YBX1	0.2481	2.101	1.650	2.674	1.657E-09
G6PD	-0.0491	1.455	1.289	1.641	1.247E-09
NOL10	0.3045	3.021	2.009	4.544	1.104E-07
DYNC1LI1	0.3166	2.638	1.907	3.649	4.593E-09
NEIL3	0.1102	1.613	1.367	1.904	1.570E-08
PTDSS2	0.2484	1.661	1.320	2.090	1.483E-05
SEPHS1	0.2446	2.513	1.815	3.479	2.813E-08
SMS	-0.0027	1.955	1.543	2.476	2.724E-08
MED8	0.1280	2.664	1.867	3.802	6.702E-08
RAMP3	-0.1211	0.674	0.573	0.793	2.124E-06

Abbreviations: HR, hazard ratio;

[a] The hazard ratio and p-value of univariate Cox proportional hazards regression analysis shown by forest plot (Figure 5D).

Table S4. Topoisomerase inhibitors, CDK inhibitors and HDAC inhibitors that predicted to transcriptionally inhibit cancer stemness of HCC.

Score [a]	Name	Target	PMID [b]
		Topoisomerase inhibitor	
-98.77	camptothecin	TOP1, HIF1A	28910823
-98.59	amonafide	TOP2A, TOP2B	32328475
-98.24	topotecan	TOP1, TOP1MT	12872448
-98.24	pidorubicine	TOP2A	8070007
-98.17	daunorubicin	TOP2A, TOP2B	10810429
-98.06	SN-38	TOP1	29031818
-97.96	idarubicin	TOP2A	24738629
-97.85	doxorubicin	TOP2A	12419750
-97.78	mitoxantrone	TOP2A, PIM1	1315428
-97.74	pirarubicin	TOP2A	30588188
-97.15	teniposide	TOP2A, CYP3A5	3039431
-97.08	irinotecan	TOP1, CYP3A5, TOP1MT	16427779
-96.58	celastrol	IL1B, TNF	25657108
-95.03	amsacrine	TOP2A, KCNH2	6286120
		CDK inhibitor	
-99.61	aminopurvalanol-a	CDK1, CDK2, CDK5, CDK6	-
-99.51	palbociclib	CDK4, CDK6, CCND3	27849562
-99.16	purvalanol-a	CDK1, CDK2, CDK4, CDK5, CCND1, CCNE1, CSNK1G3, RPS6KA1, SRC	-
-98.52	CGP-60474	CDK1, CDK2	-
-98.38	JNJ-7706621	CDK1, CDK2, AURKA, AURKB	-

-98.34	AT-7519	CDK2, CDK5, CDK1, CDK4, CDK6, CDK9	31378681
-98.31	alvocidib	CDK2, CDK4, CDK1, CDK6, CDK7, CDK9, CDK5, CDK8, EGFR, PYGM, BCL2, BIRC5, CCNT1, MCL1, XIAP	16820931
-98.27	PHA-793887	CDK1, CDK2, CDK4, CDK5, CCND1, CCNE1, CDK7, CDK9	-
-98.27	bisindolylmaleimide-ix	SIRT1, AKT1, GSK3B, LCK, LRRK2, MAPK1, MAPK11, MAPK12, MAPK14, MAPK8, PRKCA, ROCK1, RPS6KB1, SIRT2	-
-98.24	roscovitine	CDK2, CDK9, CDK7, CDK1, CDK5	21994207
		HDAC inhibitor	
-98.56	scriptaid	HDAC1, HDAC2, HDAC3, HDAC4, HDAC5, HDAC6, HDAC7, HDAC8, HDAC9	29945926
-98.36	NCH-51	HDAC1, HDAC10, HDAC11, HDAC2, HDAC3, HDAC4, HDAC5, HDAC6, HDAC7, HDAC8, HDAC9	-
-97.56	HC-toxin	HDAC1	34282273
-97.15	mocetinostat	HDAC1, HDAC2, HDAC3, HDAC11	32194803
-96.79	panobinostat	HDAC1, HDAC2, HDAC3, HDAC4, HDAC6, HDAC7, HDAC8, HDAC9	22322234
-96.48	ISOX	HDAC6	-
-96.16	apicidin	HDAC1, HDAC10, HDAC11, HDAC2, HDAC3, HDAC4, HDAC5, HDAC6, HDAC7, HDAC8, HDAC9	19376607
-95.7	vorinostat	HDAC1, HDAC2, HDAC3, HDAC6, HDAC8, HDAC10, HDAC11, HDAC5, HDAC9	31305287
-95.24	THM-I-94	HDAC1, HDAC10, HDAC2, HDAC3, HDAC6, HDAC8	-
-95.08	WT-171	HDAC6	-

[a] The result of CMap query;

[b] Experimental and clinical evidence of these compounds were searched in Pubmed.

Reference

1. Mlecnik, B., et al., *Histopathologic-based prognostic factors of colorectal cancers are associated with the state of the local immune reaction.* J Clin Oncol, 2011. **29**(6): p. 610-8.
2. Newman, A.M., et al., *Robust enumeration of cell subsets from tissue expression profiles.* Nat Methods, 2015. **12**(5): p. 453-7.
3. Bagaev, A., et al., *Conserved pan-cancer microenvironment subtypes predict response to immunotherapy.* Cancer Cell, 2021. **39**(6): p. 845-865 e7.
